# Development of Complementary Photo‐arginine/lysine to Promote Discovery of Arg/Lys hPTMs Interactomes

**DOI:** 10.1002/advs.202307526

**Published:** 2024-01-31

**Authors:** Yu Zong, Nicole Weiss, Ke Wang, Alexandra E. Pagano, Søren Heissel, Sumera Perveen, Jian Huang

**Affiliations:** ^1^ Chemical Biology Program Memorial Sloan Kettering Cancer Center New York 10065 USA; ^2^ Program of Pharmacology Weill Cornell Medical College of Cornell University New York 10065 USA; ^3^ Proteomics Resource Center Rockefeller University New York 10065 USA; ^4^ Structural Genomics Consortium University of Toronto Toronto M5S3H2 Canada; ^5^ Department of Molecular Biology Princeton University Princeton 08544 USA

**Keywords:** nucleosome chromatin remodeling, photo‐arginine, post‐translational modification, δ‐photo‐lysine

## Abstract

Arginine and lysine, frequently appearing as a pair on histones, have been proven to carry diverse modifications and execute various epigenetic regulatory functions. However, the most context‐specific and transient effectors of these marks, while significant, have evaded study as detection methods have thus far not reached a standard to capture these ephemeral events. Herein, a pair of complementary photo‐arginine/δ‐photo‐lysine (R‐dz/K‐dz) probes is developed and involve these into histone peptide, nucleosome, and chromatin substrates to capture and explore the interactomes of Arg and Lys hPTMs. By means of these developed tools, this study identifies that H3R2me2a can recruit MutS protein homolog 6 (MSH6), otherwise repelDouble PHD fingers 2 (DPF2), Retinoblastoma binding protein 4/7 (RBBP4/7). And it is disclosed that H3R2me2a inhibits the chromatin remodeling activity of the cBAF complex by blocking the interaction between DPF2 (one component of cBAF) and the nucleosome. In addition, the novel pairs of H4K5 PTMs and respective readers are highlighted, namely H4K5me‐Lethal(3)malignant brain tumor‐like protein 2 (L3MBTL2), H4K5me2‐L3MBTL2, and H4K5acK8ac‐YEATS domain‐containing protein 4 (YEATS4). These powerful tools pave the way for future investigation of related epigenetic mechanisms including but not limited to hPTMs.

## Graphical abstract

Photo‐arginine/δ‐photo‐lysine (R‐dz/K‐dz) probes are developed and involved into histone peptide, nucleosome, and chromatin to capture novel interactomes of Arg and Lys hPTMs. This work discloses that H3R2me2a inhibits the chromatin remodeling activity of the cBAF complex by blocking the interaction between DPF2 and the nucleosome. In addition, this work highlights novel pairs of H4K5 PTMs and respective readers.

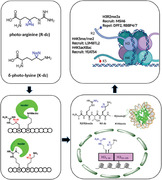

## 1 Introduction

Histone post‐translational modifications (hPTMs) constitute a major epigenetic mechanism that regulates chromatin structure and gene expression in eukaryotes.^[^ ^1^ ^]^ Of all the hPTMs, lysine (Lys/K) and arginine (Arg/R) are frequently modified, which together account for 56% and 71% of total PTMs on histones H3 and H4 respectively.^[^ ^1^, ^2^ ^]^ Lys and Arg residues commonly exhibit diverse post‐translational modifications. For example, Lys methylation (mono‐, di‐, and tri‐), acetylation, lactylation, and ubquitination have been well characterized.^[^ ^3^ ^]^ Arg is also a common site for mono methylation (Rme), asymmetric/symmetric dimethylation (Rme2a/Rme2s), and citrullination (Cit).^[^ ^4^ ^]^ Altogether, these PTMs exert exquisite regulation via recruiting or repelling specific interacting proteins, which enact downstream function. Therefore, it is of great importance to systematically explore the protein interacting profiles of these PTMs. Unlike lysine acetylation (Kac) and arginine citrullination (Cit), methylation of Lys or Arg does not alter the effective charge of residues, but rather affects the hydrophobicity and steric properties of each residues.^[^ ^5^, ^6^ ^]^ So far, a wide range of readers have been reported to recognize methylated Lys (Kme), such as Tudor, PHD, chromo, MBT, and PWWP domains. However, the effectors or readers for Arg methylation (Rme) remains largely elusive, as only Tudor domains have been shown to associate with Rme2 in particularly arginine‐ and glycine‐rich sequences.^[^ ^7^ ^]^

In general, PTM deposition and eviction are highly dynamic biochemical processes that mediate weak and transient protein‐protein interactions.^[^ ^8^ ^]^ These characteristics of PTMs have long hindered the exploration of novel direct binding partners for PTMs, including Kme and Rme. The diazirine moiety (hereafter referred as “dz”) is a common photo‐crosslinking group that serves as a powerful tool to capture protein‐protein interactions (PPIs) via irreversible chemical linkages, especially for those weak and transient interactions.^[^ ^9^, ^10^, ^11^, ^12^ ^]^

**Figure 1:**
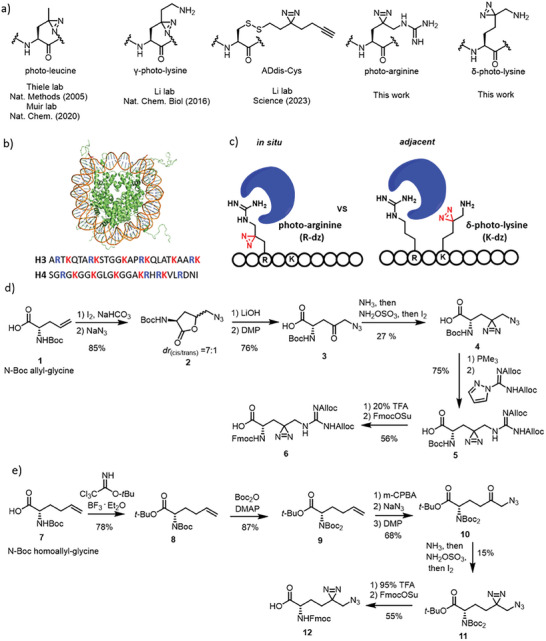
Application and synthesis of photo‐arginine and δ‐photo‐lysine probes. a) Prevalent photo‐amino acid probes for capturing the readers for PTMs of lysine. b) K/R are labeled on N‐terminus of histones H3 and H4. c) Cartoon for the in situ or adjacent capture of interacting protein. d) Synthetic route of photo‐arginine (R‐dz). e) Synthetic route of δ‐photo‐lysine (K‐dz).

Till now, four typical photo‐amino acid probes have been developed to capture the readers for Lys methylation (**Figure** **1**a). These are no doubt powerful tools to study Lys methylation which can be further modified to expand the application. For γ‐photo‐lysine, the diazirine quaternary center may make its residue side chain more rigid potentially interfering with the interactions between Kme and its binding partners. Furthermore, the inconvenient synthesis of different diazirine‐labeled probes presents an extra challenge for the systematic profiling of diverse lysine methylation states (photo‐Kme1/me2/me3). The bulky side chain of Addis‐Cys potentially restricts its applications in broad substrates. Given that leucine is not commonly found near lysine residue on histones, this approach calls for additional mutation in the vicinity of the methylated lysine for deposition of the neighboring photo‐leucine. Moreover, to the best of our knowledge, no chemical tools to investigate the PTMs of arginine have been reported.

Herein, we develop a pair of complementary photo‐arginine/ δ‐photo‐lysine (R‐dz/K‐dz) tools to explore the interactomes of Arg and Lys hPTMs. We discover that H3R2 asymmetric methylation (H3R2me2a) can recruit MSH6 and repel DPF2/ RBBP4/RBBP7 at the peptide, nucleosome and chromatin level. By means of these powerful tools, we reveal that H3R2me2a hinders the chromatin remodeling function of cBAF by impeding the binding between DPF2 (a constituent of cBAF) and the nucleosome. In the meantime, we methodically emphasize the novel combinations of Histone H4 PTMs and “readers”, such as H4K5me‐L3MBTL2, H4K5me2‐L3MBTL2, and H4K5acK8ac‐YEATS4 individually. These findings lay the groundwork for future exploration of the corresponding epigenetic mechanisms involving histone H4.

## 2 Results and Discussion

### 2.1 Generation of Photo‐Arginine/ δ‐Photo‐Lysine (R‐dz/K‐dz) Building Blocks

In situ, photo‐lysine and adjacent photo‐leucine have been developed for studying PTMs on lysine.^[^ ^9^, ^10^ ^]^ As of yet, there are no known chemical tools that are capable of exploring the PTMs of arginine residues. From the insights gained through the investigation of lysine PTMs, when studying PTMs on arginine residues, the initial step is determining whether the diazirine deposits should be positioned in situ or in close proximity to the PTM site to best emulate the native peptide. Upon inspecting the sequence of histones, it is evident that Arg and Lys commonly appear as a pair (Figure 1b). Logically, we determine to generate in situ diazirine‐labeled (R‐dz) and adjacent diazirine‐labeled (K‐ dz) peptides to address this question (Figure 1c). To this end, we set out to establish concise pathway to build up the key photo probes, R‐dz and K‐dz.

To synthesize the photo‐arginine (R‐dz) probe, compound 2 was prepared via iodine‐mediated cyclization of the commercially available N‐Boc‐allylglycine, followed by the substitution of NaN_3_.^[^ ^13^ ^]^ After sequential hydrolysis by Lithium hydroxide (LiOH), and oxidation by Dess–Martin periodinane (DMP) separately, the γ‐keto was introduced on compound 3. The keto was then converted to diazirine via sequentially adding ammonia, hydroxylamine‐O‐sulfonic acid (HOSA), and iodine to obtain compound 4. Next, the azide group was reduced by PMe_3_, followed by guanidination to form compound 5. After the removal of Boc and reprotection with Fmoc, compound 6 which is ready for solid‐phase peptide synthesis (SPPS) was generated (Figure 1d).

For the design of diazirine‐labeled Lys, there are two comparative positions, γ and δ, which may accommodate the diazirine. Li group achieved the first synthesis of γ‐photo‐lysine over twenty steps.^[^ ^9^ ^]^ Based on the synthetic route of photo‐arginine, it could immensely simplify the synthetic pathway to deposit diazirine group at δ position of lysine. Considering the challenge of achieving 6‐exo cyclization, we adopted another strategy to obtain γ‐photo‐lysine (K‐dz) via the difunctionalization of alkene. To avert the side cyclization initiated by NHBoc or free carboxylic acid, initially, α‐NH and carboxylic acid of compound 7 were protected to generate compound 9. Then the compound 9 experiencing sequential epoxidation, substitution, and oxidation was turned to intermediate 10. According to the same procedure to access compound 4, diazirine was installed on compound 11. At last, after removal of Boc/*t*Bu and reprotection with Fmoc, final compound 12 applicable for SPPS was obtained (Figure 1e). Given that these two probes are newly prepared, we explored the labeling characteristic of photo‐arginine. Woo et al. reported that positively charged probes had higher labeling propensities that may reflect increased interaction with negatively charged protein surface.^[^ ^14^ ^]^ Herein, we explored which kinds of amino acids photo‐arginine preferred to label. We selected six presentative amino acids encompassing positive charge, negative charge, and neutral amino acids (Asp, Trp, Lys, Tyr, Ile, and Gln). We observed that photo‐arginine preferred to label Asp (negative charge) and Lys (the carboxylic acid of Arg interacting with side chain of Lys). In addition, Photo‐arginine also preferred to label Trp and Tyr which potentially could form Cation‐Π interaction with Arg. The neutral amino acids Ile and Gln exhibited relatively lower labeling efficiency (Figure S1, Supporting Information).

### 2.2 Exploration of the Interactomes of H3R2me2a/H3R2

**Figure 2:**
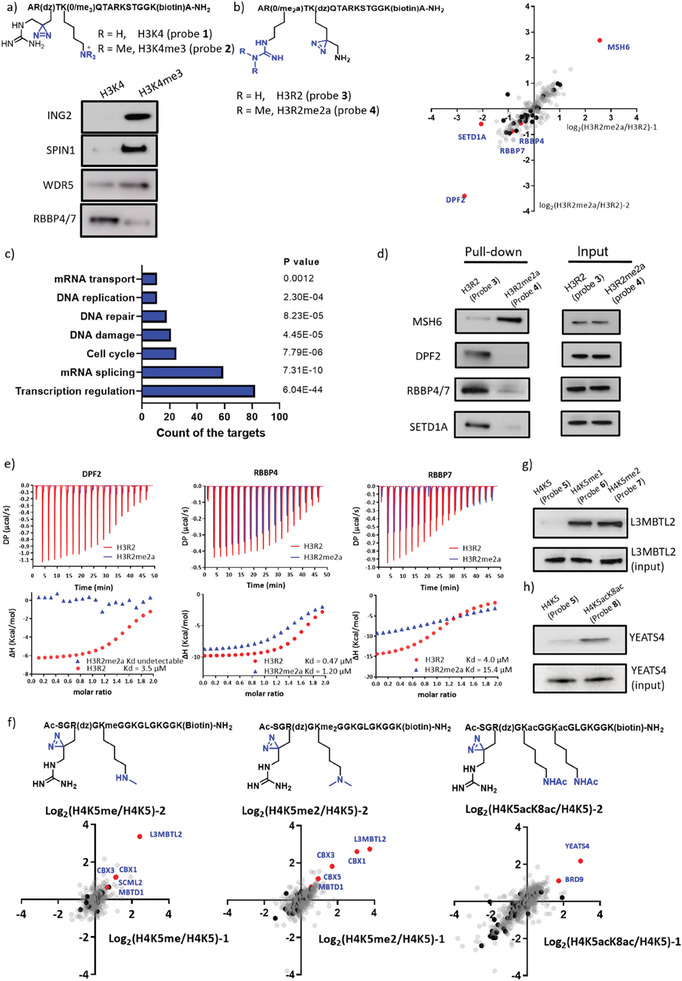
Identification of interactomes of hPTMs on H3/H4. a) Western blot analysis of pull‐down targets by H3K4 (probe 1) and H3K4me3 (probe 2) peptides. n =  2, with representative data shown. b) SILAC‐based proteomic analysis of H3R2me2a/H3R2 (histone binding proteins were highlighted by black and red dots). n = 2. c) Biological process analysis of H3R2/H3R2me2a dataset in Figure 2b. d) Western blot analysis of pull‐down targets by H3R2 (probe 3) and H3R2me2a (probe 4) peptides. n =  2, with representative data shown. e) ITC measurement for the binding affinities of native H3R2/H3R2me2a peptides (H3_1‐15_ without diazirine and biotin) and DPF2_PHD_/RBBP4/RBBP7. n =  2, with representative data shown. f) SILAC‐based proteomic analysis of H4K5me1/H4K5, H4K5me2/H4K5, and H4K5acK8ac/H4K5 (histone binding protein were highlighted by black and red dots). n =  2. g) Western blot analysis of pull‐down targets by H4K5/me1/me2 peptides. n =  2, with representative data shown. h) Western blot analysis of pull‐down targets by H4K5/H4K5acK8ac peptides. n =  2, with representative data shown.

With photo‐arginine and δ‐photo‐lysine in hand, we initially demonstrated the robustness of our photo‐arginine probe with well‐investigated H3K4me3 modification. We generated two probes H3K4‐R2dz (probe 1) and H3K4me3‐R2dz (probe 2) and pulled the target protein from HEK 293T nuclear lysate for western blot analysis. SPIN1, ING2, WDR5 and RBBP4/7 were established target for H3K4/H3K4me3.^[^ ^9^, ^10^ ^]^ Herein, we found SPIN1, ING2 and WDR5 were enriched in H3K4me3 group, and RBBP4/7 were enriched in H3K4 group. These findings aligned well with established results (**Figure** **2**a). Prior research has shown that H3R2me2a and H3K4me3 are mutually exclusive markers at euchromatic genes.^[^ ^15^ ^]^ H3R2me2a, deposited by PRMT6 has been associated with gene repression at promoters and gene activation at enhancers.^[^ ^16^ ^]^ The opposing effects of H3R2me2a on gene regulation may correlate with the recruitment or the repulsion of different binding partners, making this an attractive mark for investigation. Prior to determining the downstream interactomes of H3R2mea using a photo‐crosslinking (diazirine) peptide, we first assessed whether the diazirine moiety, when situated in situ (H3R2‐dz) or adjacent (H3K4‐dz), would more closely resemble the native H3R2 peptide. Alongside the diazirine group, a pre‐existing biotin moiety (handle for pull‐down) was also incorporated to assess the binding affinity (Figure S2, Supporting Information). Given the PHD domain of UHRF1 (UHRF1_PHD_) could interact with N terminus of H3,^[^ ^17^ ^]^ we quantitatively evaluated the binding affinity between UHRF1_PHD_ and H3R2‐dz/ H3K4‐dz via Isothermal titration calorimetry (ITC) assay. The Kd of H3R2, H3R2‐dz, and H3K4‐dz were 2.3, 10.6, and 6.4 µm separately (Figure S2, Supporting Information). It indicated H3K4‐dz could better mimic native peptide H3R2 and the influence of pre‐installed biotin for binding affinity was negligible.

We speculated that the in situ diazirine on arginine generated a quaternary carbon center and made the side chain slightly more rigid to interfere the interaction between R2 and UHRF1_PHD_. Next, probes 3 and 4 were exploited to systematically probe the interactomes of H3R2 and H3R2me2a in HEK293T nuclear lysates via SILAC‐based quantitative proteomic analysis (Figure 2b). Then we conducted the bioinformatic analysis based on the SILAC‐based quantitative proteomic analysis of H3R2/H3R2me2a (https://david.ncifcrf.gov/tools.jsp). There were over 50 chromatin‐binding proteins and over 30 histone‐binding proteins enriched in H3R2/H3R2me2a dataset (Table S1, Supporting Information). As we know, histone PTMs were widely involved in genome topology, DNA repair, transcription, RNA splicing, and so on.^[^ ^2^, ^18^ ^]^ In the meanwhile, we analyzed the biological process based on H3R2/H3R2me2a dataset, and found that ≈80 and 60 target proteins participated in transcription regulation and mRNA splicing respectively (Figure 2c). These bioinformatic analysis suggested that the quality of identification was satisfactory. Since it is the first time to systematically investigate direct interacting protein for arginine methylation (H3R2me2a), we focused on the 32 histone binding protein (highlighted by black and red dots in Figure 2b). Initially, we showed that H3R2me2a modification sufficed to repel SETD1A, a histone methyltransferase to deposit H3K4me3 (Figure 2b). As mentioned, H3R2me2a and H3K4me3 are mutually exclusive at euchromatic genes.^[^ ^12^ ^]^ This observation indicated a complementary mechanism for the antagonizing effects of H3R2me2a on H3K4me3. RBBP4/7, as an indispensable part of the Polycomb repressive complex (PRC2)^[^ ^19^ ^]^ and Nucleosome Remodeling and Deacetylase (NuRD) complex,^[^ ^20^ ^]^ takes part in the deposition of H3K27me3 mark as well as the deacetylation of H3K27ac respectively. Herein, we found R2me2a methylation could decrease the interaction between RBBP4/7 and Histone H3. Previously, H3K4me3 could inhibit the interaction between H3 and Nurf55 (Drosophila RBBP4/7)‐Suz12, and further inhibit PRC2 activity.^[^ ^21^ ^]^ Additionally, the interaction between RBBP4 and H3 tails has been demonstrated to be essential for the recruitment of NuRD complex to the chromatin.^[^ ^20^ ^]^ Hence, H3R2me2a could potentially impede the deposition of H3K27me3 and the deacetylation by attenuating the interaction of H3 and RBBP4/7. It is worth mentioning that DPF2 is completely repelled, whereas MSH6 is most significantly recruited by H3R2me2a (Figure 2b). It was reported that PWWP domain of MSH6 could recognize the H3K36me3 mark and was recruited to the chromatin for DNA mismatch repair (MMR).^[^ ^22^ ^]^ Additionally, based on the matched sequence of H3R2me2a signal and MMR events (G1 to early S phase),^[^ ^23^ ^]^ we reasoned that H3R2me2a deposited by PRMT6 provided an alternative way to engage MSH6 for MMR. DPF2, as a key component of cBAF, was reported to bind the N‐terminus of histone H3 and participate in the cBAF chromatin remodeling activity.^[^ ^24^, ^25^ ^]^ We speculated H3R2me2a could impede the cBAF activity via blocking the interaction between histone H3 and DPF2. To avoid following the false positive results, we checked the MS/MS signaling of these promising targets (Figure S3, Supporting Information) and western blot analysis of the pull‐down targets by H3R2 and H3R2me2a probes were consistent with the results from proteomic dataset (Figure 2d).

Given that the aforementioned target proteins (DPF2, RBBP4/7, and MSH6) influenced by H3R2me2a are appealing, we embarked on quantitative evaluation of the variation of binding affinity caused by methylation. First, we ascertained the binding domains of these target protein. Nurf55 (Drosophila RBBP4/7)^[^ ^19^ ^]^ and tandem PHD domain of DPF2 (DPF2_PHD_)^[^ ^24^, ^25^ ^]^ have been reported to bind the N‐terminus of histone H3. In addition, the PWWP domain of MSH6 (MSH6_PWWP_) was shown to bind H3K36me3.^[^ ^22^ ^]^ Considering that known reader of Kme3 may also recognize Rme2a,^[^ ^26^ ^]^ we speculated that MSH6_PWWP_ may recognize H3R2me2a. We purified these domains and synthesized the associated native H3 peptide (H3_1‐15_ without diazirine and biotin) for quantitative evaluation via ITC. The Kd for DPF2_PHD_ and the H3R2 peptide was 3.5 µm, however there was no binding affinity detected between DPF2_PHD_ and the H3R2me2a peptide (Figure 2e). As to RBBP4 and RBBP7, the Kd values for H3R2 binding were found to be 0.47 and 4.0 µm respectively, while binding for H3R2me2a were ≈1.2 and 15.4 µm, indicating that asymmetric methylation resulted in three–fourfold decrease in binding affinity (Figure 2e). However, the weak interaction between MSH6_PWWP_ and H3R2me2a was undetectable via ITC. Even so, the MSH6_PWWP_ domain was more efficiently covalently labeled by the H3R2me2a than by the H3R2 probe in vitro (Figure S4, Supporting Information). Given that MSH6 possesses more than one domain, the other domains, such as the DNA mismatch binding domain may also contribute to the binding affinity for H3. Notably, this coincidently highlights the advantage of our diazirine‐labeled probes as they are better at capturing and differentiating these weak protein‐protein interactions. As mentioned, Li group reported the first useful γ‐photo‐lysine to capture the readers for H3K4me3.^[^ ^9^ ^]^ However, the lengthy synthetic pathway (over 20 steps) potentially restrict its broad applications. Compared with the route to synthesize γ‐photo‐lysine, our synthetic route (5‐step to get photo‐arginine and δ‐photo‐lysine) is shorter and easier for applications. Photo‐leucine is another well‐developed and commercially available tool. Compared with photo‐leucine probe, our δ‐photo‐lysine shows higher cross‐linking efficiency and lower labeling background (Figure S5, Supporting Information).

### 2.3 Exploration of the Interactomes of H4K5me1/me2 and H4K5acK8ac

K‐dz has been verified to be a useful tool to explore the effect of arginine methylation, and considering that Lys and Arg appear as a pair on histones, photo‐arginine (R‐dz) would also be a powerful tool to trap the interactomes of PTMs on lysine. Considering that H3 modifications at H3K4, H3K9, and H3K27 have been relatively well‐characterized, our study focused on exploring the hPTMs of histone H4 using photo‐arginine probe. Since the N‐terminus sequence of H4 was flat, R‐dz would be the primarily preferred option to study Lys PTMs on H4 N‐terminus. H4K5me and H4K5acK8ac are well‐established modifications separately.^[^ ^27^, ^28^ ^]^ To understand the downstream effects generated by these different PTMs, specific readers remain to be identified and characterized in respect to each mark. To further expand the application of photo‐arginine probe, we synthesized the related peptides H4K5, H4K5me, H4K5me2, and H4K5acK8ac (probes 5–8), and conducted SILAC‐based quantitative proteomic analysis (Figure 2f). After SILAC‐based quantitative proteomic analysis, we first conducted the GO analysis to collect all histone binding protein (labeled with black and red dots) (Figure 2f; Table S1, Supporting Information). Within the histone binding protein category, our focus was directed toward target proteins that possess reader domains relevant to our study. Within the H4K5/H4K5me group, L3MBTL2, CBX1, CBX3, SCML2, and MBTD1 possessed domains relevant to the recognition of Kme. In the H4K5/H4K5me2 group, L3MBTL2, CBX1, CBX3, CBX5, and MBTD1 similarly harbored associated domains for interpreting Kme2. Additionally, in the H4K5/H4K5acK8ac group, YEATS4 and BRD9 stood out as the top two known candidates for recognizing Kac. we discovered that both H4K5me and H4K5me2 could recruit L3MBTL2, while YEATS4 was enriched in H4K5acK8ac dataset. These results were also confirmed by western blot analysis (Figure 2g,h). Furthermore, it was noted that the augmentation of CBX1/3 occurred concomitantly with the progression of methylation levels (Figure 2f). Given the established role of CBX1/3 in recognizing H3K9me2/me3,^[^ ^29^ ^]^ it is reasonable to hypothesize that CBX1/3 might also serve as potential readers for H4K5me3. BET family has been reported to recognize Kac.^[^ ^28^ ^]^ In this study, besides L3MBTL2, we discovered that BRD9 also exhibited enrichment within the bivalent modification H4K5acK8ac group. L3MBTL2 preferred to bind H4K20me1/me2.^[^ ^30^ ^]^ Herein, we found H4K5me1/me2 provided alternative way to recruit L3MBTL2 to exert downstream functions. YEATS4 is frequently amplified in human non‐small cell lung cancer (NSCLC) and required for cancer cell proliferation, survival, and transformation.^[^ ^31^ ^]^ Furthermore, dimeric YEATS4 can recognize bivalent modification of H3K23acK27ac.^[^ ^32^ ^]^ YEATS4 as newly identified reader for H4K5acK8ac is worth exploring its co‐localization with H4K5acK8ac mark on chromatin and seeking new pathway regulated by H4K5acK8ac.

### 2.4 Validation of the Interaction at Nucleosome and Chromatin Level

**Figure 3:**
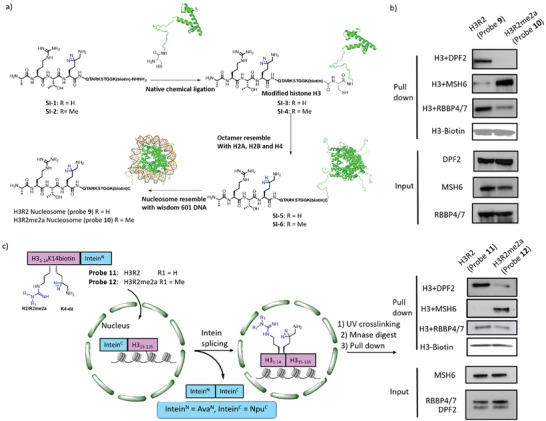
Validation of interactomes of hPTMs at nucleosome and chromatin level. a) Workflow to generate the mononucleosome H3R2 probe 9 (H3R2‐K4dz‐K14biotin) and H3R2me2a probe 10 (H3R2me2a‐K4dz‐K14biotin). b) Western blot analysis of probe 9 and probe 10 pull‐down interacting protein. c) Schematic for the capture of interacting protein via trans‐splicing of H3R2 (probe 11)/H3R2me2a (probe 12) on chromatin. Western blot analysis of interacting protein enriched by H3R2 (probe 11)/H3R2me2a (probe 12). n =  2, with representative data shown.

After evaluating and elucidating how H3R2me2a recruits and repels target proteins at the peptide level, we moved forward to exploit diazirine‐labeled nucleosome to validate the direct interaction between potential targets and H3R2me2a in a more complex and biologically relevant system. Although the amber suppression system has been well‐established to incorporate diazirine‐labeled lysine on nucleosome,^[^ ^33^, ^34^ ^]^ it remains impossible to incorporate methylated Arg due to the absence of an appropriate suppressor tRNA/ amino acyl‐tRNA synthetase. Therefore, we combined semi‐synthesis and native chemical ligation strategy to incorporate R2me2a (PTM), K4‐dz (photo crosslinker), and K14biotin (affinity handle for pull‐down) into nucleosomes (**Figure** **3**a; Figure S6, Supporting Information). After covalent labeling and pull‐down with diazirine‐labeled mononucleosome from HEK293T nuclear lysate, MSH6 linking H3 (H3+MSH6) was enriched in the H3R2me2a (probe 10) group, the adducts of H3+DPF2 and H3+RBBP4/7 were repelled by the R2me2a (Figure 3b). After validation with nucleosome, we were curious about how these targets proteins interact with H3R2me2a at the chromatin level in intact nuclei. Previously, intein^N^‐intein^C^ (Ava^N^‐Npu^C^) split intein system was developed to incorporate various non‐native chemical modifications into chromatin.^[^ ^35^ ^]^ Recently, split intein system has been proven to be applied to study the interactomes of lysine modification on H3.^[^ ^10^ ^]^ We decided to utilize the Ava^N^‐Npu^C^ split intein system to further validate the interactions of potential targets and H3R2/R2me2a at the chromatin level in nuclei (Figure 3c). Initially, we confirmed that the C‐terminus Npu^C^ fused with H3 (Npu^C^‐H3^C^_15‐135_) could incorporate into chromatin. Meanwhile, the N‐terminus of H3 linking N‐terminus Ava^N^ (H3^N^_1‐14_‐Ava^N^) could react with Npu^C^‐H3^C^_15‐135_ to generate intact H3 on chromatin (Figure S7, Supporting Information). After removal of excessive H3^N^_1‐14_‐Ava^N^ and UV crosslinking, target proteins were irreversibly attached on chromatin. After sequential chromatin digestion, target enrichment, and western blot analysis, MSH6 was enriched whereas DPF2 and RBBP4/7 were repelled by R2me2a (probe 12) at the chromatin level (Figure 3c).

### 2.5 H3R2me2a Inhibits cBAF Activity Via Repelling DPF2

**Figure 4:**
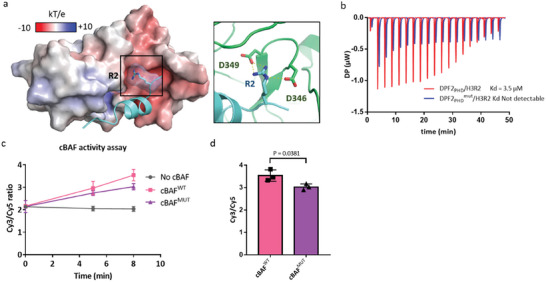
H3R2me2a inhibits cBAF activity via repelling DPF2. a) Structural modeling of DPF2_PHD_ and H3R2 peptide, and D346/349 show hydrogen bonds with guanidine group of H3R2. b) ITC measurement for the binding affinity of DPF2_PHD(D346/349A)_ and native H3_1‐15_ peptide. n =  2, with representative data shown. c) In vitro test of chromatin remodeling activity of cBAF (DPF2^WT^) and mutant cBAF (DPF2^D346/349A^) complex with nucleosome substrate (Cy5 labeled on histone H2A and Cy3 labeled on template DNA). Data represents the mean±SD of Cy3/Cy5 ratio from three biological replications n = 3. d) Presentation of chromatin remodeling activity of cBAF^WT^ and cBAF^MUT^ at 8 min from Figure 4c. P value is generated by student's *t*‐test.

As reported, H3R2me2a resulted in a two‐fold reduction in cBAF chromatin remodeling activity relative to unmodified mononucleosome, with no reduction in PBAF and ncBAF activity.^[^ ^36^ ^]^ DPF2, as a key component of cBAF, not involved in PBAF and ncBAF, was repelled from H3R2me2a nucleosome (Figure 3b,c). Hence, we proposed that DPF2 played a key role in mediating the correlation between H3R2me2a mark and cBAF activity. To address the detailed interaction between H3R2me2a and DPF2, we need to understand how DPF2 interacts with native H3. Li lab reported the initial discovery of an interaction between DPF2 and H3.^[^ ^24^ ^]^ They indicated that obtaining a co‐crystal structure of DPF2 and H3 was likely a challenging endeavor. Considering the absence of a reported co‐crystal structure of DPF2 and H3, we docked H3 peptide against DPF2 in accordance with the co‐crystal structure of DPF3_PHD_ (80% sequence identity to DPF2_PHD_) and H3.^[^ ^37^ ^]^ Based on the docking results, we found the free guanidine moiety of R2 formed hydrogen bonds and salt bridge with the carboxylic acids of both D346 and D349 in DPF2 (**Figure** **4**a). Due to the increased steric properties and missed hydrogen bond, asymmetric R2me2a could not reside into the pocket formed by D346/ D349. To futher emphasize the contribution of D346/ D349 to cBAF activity, we generated double mutates (D346/349A) in DPF2. Initially, the Kd between the PHD domain of DPF2D346/349A and H3 could not be detected, which indicated that the mutated DPF2 was unable to bind to histone H3 as efficiently as it did previously (Figure 4b). As aforementioned, compared with H3R2 nucleosome, the H3R2me2a inhibited the cBAF activity.^[^ ^36^ ^]^ Then we generated the lentivirus for HA‐DPF2^WT^ and HA‐DPF2 ^D346/349A^ to transduce HEK293T cells to express HA‐DPF2^WT^ and HA‐DPF2 ^D346/349A^ separately. Subsequently, we purified the complexes of cBAF^WT^ (HA‐DPF2^WT^) and cBAF^MUT^ (HA‐DPF2^D346/349A^) and utilized them to assess their chromatin remodeling activity in vitro (Figure S8, Supporting Information). Compared with cBAF (DPF2^WT^), mutant cBAF (DPF2^D346/349A^) complex showed decreased remodeling activity (Figure 4c,d). It indicated that inhibition of cBAF activity with H3R2me2a nucleosome was due to the impediment of DPF2 recruitment. Previously, DPF2 was reported to repress myeloid differentiation;^[^ ^25^ ^]^ Otherwise, PRMT6 exhibited an inverse effect to promote myeloid differentiation.^[^ ^38^ ^]^ Considering the exclusive interaction of DPF2 and H3R2me2a (deposited by PRMT6) marked nucleosome, in the future, it deserves to explore the repressive role in myeloid differentiation mediated by DPF2 can be blocked by H3R2me2a.

## 3 Conclusion

In summary, we elaborately developed a pair of scalable photo‐arginine (R‐dz) and δ‐photo‐lysine (K‐dz) probes for systematic assessment of arginine and lysine hPTMs; R‐dz and K‐dz were incorporated in peptide, nucleosome, and chromatin substrates to profile dynamic interactomes of Arg/Lys PTMs; For H3R2me2a, We elucidated the molecular mechanism underlying the repulsion of DPF2 by H3R2me2a, which subsequently impeded the chromatin remodeling activity of cBAF; For H4K5 PTMs, we discovered L3MBTL2 acted as the reader for H4K5me1/me2, while YEATS4 functioned as the reader for H4K5acK8ac. Recently, more new PTMs (such as H3Q5ser, H3P16oh, and H4K5la) are identified and play essential role in regulating cellular signaling.^[^ ^39^, ^40^, ^41^ ^]^ The neighbor R‐dz and K‐dz will be primarily preferred to study their interactomes. Given the broad applicability of R‐dz and K‐dz tools, their usage extends beyond the identification of histone K/R PTMs. These tools are poised to play a crucial role in profiling the interactome of non‐histone PTMs where either lysine or arginine is proximal. This potential application holds promise for the future.

## 4 Experimental Section

### Peptide Synthesis and Purification

All of the peptides were synthesized at a 0.02 mmol scale by standard Fmoc‐based solid‐phase peptide synthesis. Resin was Rink amide ProTide (loading: 0.58 mmol g^−1^). For the probes 1–4, C‐terminus of QTARKSTGGK(biotin)A was synthesized using DIC‐Oxyma activation on a CEM Liberty Blue microwave‐assisted peptide synthesizer. N‐terminus of AR(dz)TK, AR(dz)TKme3, ARTK(dz), and ARme2aTK(dz) were coupled for 1 h using HATU/DIPEA as coupling reagents at room temperature. For peptides 5–8, C‐terminus of GKGGKGLGKGGK(biotin) was synthesized using DIC‐Oxyma activation on a CEM Liberty Blue microwave‐assisted peptide synthesizer, R(dz) was coupled using DIC/HOBT at room temperature overnight, N‐terminus of SG was coupled using HATU/DIPEA at room temperature. For the deprotection of azide group on the precursor of K‐dz, the resin (0.01 mmol) was dissolved in 1 mL THF/H_2_O (20:1), then 100 µL of 1 m PMe_3_ was added and the reaction was conducted under the N_2_ atmosphere at room temperature for 15 min, the azide group (‐N_3_) was reduced to amine (−NH_2_). For the deprotection of alloc group, the resin (0.01 mmol) was dissolved in 1 mL anhydrous DCM, 50 µL phenylsilane, and 6 mg of Pd(PPh_3_)_4_ was added, the reaction was stirred for 30 min at room temperature under Ar atmosphere. For the deprotection of all acid‐labile groups, the resin was dissolved in 2 mL cleavage cocktail (TFA: TIPS: DODT: H_2_O/ 92.5:2.5:2.5:2.5) and was stirred for 1 h at room temperature. Then the volume of cleavage solution was reduced to less than 200 µL and the crude peptide was precipitated with cold diethyl ether. Preparative scale RP‐HPLC was performed on Waters 2545 instrument equipped with a Waters xBridge Prep C18 5 µm OBD column (19 × 150 mm) at a flow rate of 15 mL min^−1^. The mobile phase comprised 0.1% TFA in water (solvent A) and 0.1% TFA in acetonitrile (solvent B). The program is 0–20% B gradient in 30 min.

### C‐Terminal Hydrazides Peptide Synthesis and Purification

For peptides SI‐1 and SI‐2, commercially available 2‐chlorotrityl chloride resin (2.7 g, substitution: 0.37 mmol g^−1^) was swelled in DCM for 15 min. Fmoc‐NHNH_2_ (1.02 g, 4 mmol) and DIEA (1.65 mL, 10 mmol) in DMF were added into the resin at 0 °C. The reaction mixture was gradually warmed to ambient temperature and stirred overnight. After completion, methanol was added, and the solution was stirred for 10 min to block out the activated chlorine group. Finally, the resin was filtered and washed with DMF, methanol, DCM, and diethyl ether. After that, the coupling procedure of probes SI‐1 and SI‐2 refers to the synthesis of probes 3 and 4; The native peptides were synthesized using DIC‐Oxyma activation on a CEM Liberty Blue microwave‐assisted peptide synthesizer.

### Stable Isotope Labeling of Amino Acids in Cell Culture (SILAC) and SILAC‐Based Pull‐Down Assay

HEK293T cells were plated in DMEM for SILAC media (88364, Thermo Scientific), supplemented with 10% dialyzed FBS (16777‐212, VWR), 1x penicillin–streptomycin (diluted from 100x Pen Strep, 15‐070‐063, Thermo Fisher), and “heavy” isotope labeled (^13^C_6_–^15^N_2_–Lys and ^13^C_6_–^15^N_4_–Arg) (88209, 89990, Thermo Fisher) or “light” Lys and Arg (89 987, 89 989, Thermo Fisher). After complete isotopic labeling, the cells were harvested separately to get the cell pellet. Cell pellet was washed with cold PBS three times to remove FBS in the following steps. 8 × 10^7^ cells was used for each group pull‐down. Then the nuclear extraction was collected with NE‐PEP Nuclear and Cytoplasmic Extraction Reagents (Thermo Scientific 78 835). The extraction of nuclear was diluted with cytoplasmic extraction reagent I (two volume of nuclear extraction reagents). The final concentration of protein in diluted nuclear extraction buffer is 0.5 mg mL^−1^ and the volume is 1 mL. The heavy isotope‐labeled group and light isotope‐labeled group were treated with 10 µµ H3R2 (probe 3) and H3R2me2a (probe 4) respectively and incubated at 4 °C for 1 h. Then they were placed on ice and irradiated with 365 nm UV light for 30 min. After irradiation, the heavy and light group were mixed and 8 mL cold MeOH was added and the protein was precipitated at −20 °C overnight. Then the protein pellets were centrifuged at 4000 rpm for 30 min at 4 °C, pellets were transferred to 2.0 mL centrifuge tube and pellets were let air dried (37° for 10 min) before being re‐solubilized in 250 µL 4% SDS PBS with bath sonication. Solutions were diluted with 750 µL PBS, and incubated with 50 µL High Capacity streptavidin agarose resin (thermo scientific 20 359) at room temperature for 1 h with end‐to‐end rotation. Agarose was washed with 500 µL 1% SDS PBS three times, 500 µL 4 m Urea PBS three times, and 500 µL PBS three times. Partial trypsin digestion was used to elute proteins from beads. Eluted proteins were reduced, alkylated, and digested a second time with trypsin and lysC. Peptides were cleaned‐up using solid‐phase extraction. Samples were analyzed using an orbitrap Fusion‐LUMOS tribrid mass spectrometer (Thermo Scientific) operated in high/high mode and peptides separated using a 90 min gradient (not counting loading and washing). Spectra were queried against a human database concatenated with common contaminants allowing for identification and quantification of heavy lysine (Lys8) and arginine (Arg10) using the MASCOT search engine. Matched peptides were filtered to only include matches w/FDR of 1% or better. Data analysis was performed using Proteome Discoverer v. 1.4 (Thermo Scientific). Abundance values were log_2_ transformed and normalized to the median intensity. Proteins identified from only one peptide were excluded. Heavy isotope labeled HEK293T cell was used for H3R2me2a (probe 4), H4K5me (probe 6), H4K5me2 (probe 7), and H4K5acK8ac (probe 8); and Light isotope labeled HEK293T cell was used for H3R2 (probe 3) and H4K5 (probe 5).

### General Pull‐Down Procedure by Diazirine Labeled Peptides

The protocol refers to SILAC‐based pull‐down assay with the following modifications. HEK293T cells were plated in normal DMEM medium and the protocol of nuclear lysate extraction is as same as SILAC‐based assay. After incubating and crosslinking with 10 µµ peptides probes, the whole protein in each group was precipitated and resolved in 1% SDS PBS buffer. Then peptides trapped protein was enriched with 50 µL high‐capacity streptavidin agarose resin separately. The enriched protein on beads were released in 4X Laemmli sample buffer by heating at 98 °C for 20 min. At last, the samples were run on a 4–12% bis‐tris SDS‐PAGE gel and analyzed by western blot. The assay was done two times to confirm results.

### Expression and Purification of Recombinant Proteins: RBBP4/7

DNA fragment encoding RBBP4/7 (1‐ 425) was cloned into pFBOH‐LIC donor plasmid. The resulting plasmid was transformed into DH10Bac Competent E. coli cells (Invitrogen) and a recombinant Bacmid DNA was purified and followed by a recombinant baculovirus generation in sf9 insect cells. sf9 cells grown in HyQ SFX insect serum‐free medium (Thermo Scientific) were infected with 10 mL of P3 viral stocks per 1 L of suspension cell culture and incubated at 27 °C using a platform shaker set at 150 rpm. The cells were harvested by centrifugation (4000 rpm for 10 min at 4 °C) after 72 h of post‐infection time when viability dropped to 70–80%. Harvested cells were re‐suspended in 20 mm Tris‐HCl, pH 8.0, 500 mm NaCl, 5 mm imidazole, and 5% glycerol, 1X protease inhibitor cocktail (100 X protease inhibitor stock in 70% ethanol (0.25 mg mL^−1^ Aprotinin, 0.25 mg mL^−1^ Leupeptin, 0.25 mg mL^−1^ Pepstatin A, and 0.25 mg mL^−1^ E‐64) or Pierce Protease Inhibitor Mini Tablets, EDTA‐free. The cells were lysed chemically by rotating for 30 min after addition of 1 mm PMSF, 1 mm TCEP, 0.5% NP40, and 15 µL Benzonase Nuclease (In‐House) followed by sonication at frequency of 7 (5″ on/7″ off) for 2 min (Sonicator 3000, Misoni). The crude extract was clarified by high‐speed centrifugation (60 min at 36000 ×g at 4 ˚C) by Beckman Coulter centrifuge. Clarified lysate was then incubated for ≈1 h at 4 °C with pre‐equilibrated Ni‐NTA (Qiagen) in batch adsorption format on a rotating platform. Resin was then transferred to an open gravity column (Bio‐Rad) and was washed with binding buffer followed by washing buffer containing 20 mm Tris‐HCl, pH 7.5, 500 mm NaCl, 5% glycerol, 5 mm imidazole, and 30 mm Imidazole, respectively. Protein was then eluted with 20 mm Tris‐HCl, pH 7.5, 500 mm NaCl, 5% glycerol, 250 mm imidazole. Next, the eluate was diluted 10x with Buffer A (20 mm Tris, pH 8.0) and loaded onto a Resource Q ion exchange column (GE Healthcare), and eluted over 40 CV with Buffer B (20 mm Tris‐HCl, pH 8.0, 1 m NaCl). After analysis by SDS‐PAGE, fractions from the first peak containing the protein were pooled and 1 mm TCEP was added before concentrating. To further purify the protein, it was loaded on a gel filtration HiLoad Superdex 75 16/600 (GE Healthcare) column pre‐equilibrated in 20 mm Tris‐HCl, pH 8.0, 200 mm NaCl, 5% glycerol, 1 mm TCEP. Fractions were assessed by SDS‐PAGE electrophoresis and those containing pure protein were pooled, concentrated, and flash‐frozen for storage at −80 °C.

### Expression and Purification of Recombinant Proteins: His‐DPF2_PHD_

His‐DPF2_PHD_ (residues 270–391) domain in pET28‐MHL vector. The expression and purification were conducted as described with the following modifications.^24^ His‐DPF2_PHD_ were expressed in Escherichia coli strain BL21 (DE3) (Novagen) with 0.5 mm IPTG induction at 16 °C in LB medium supplemented with 0.1 mm ZnCl_2_. After cell lysis by French Press Cell Disrupter and centrifugation, the supernatant was applied to a HisTrap (GE Healthcare) nickel column, and the bound protein was eluted with a linear imidazole gradient from 20 to 500 mm. And further purified by a HiLoad 16/60 Superdex 75 (GE Healthcare) gel filtration column. The pure protein were pooled and dialyzed in buffer (100 mm NaCl, 20 mm Tris, pH 7.5) with Slide‐A‐Lyzer (10000 MWCO). At last, the dialyzed protein were concentrated with to 5 mg mL^−1^ and stored in −80 °C freezer for future use.

### Expression and Purification of Recombinant Proteins: His‐MSH6_PWWP_

His‐MSH6_PWWP_ (residues 87–198) domain in pET28‐MHL vector. His‐ MSH6_PWWP_ were expressed in Escherichia coli strain BL21 (DE3) (Novagen) with 0.5 mM IPTG induction at 16 °C in LB medium. After cell lysis by French Press Cell Disrupter (Thermo) and centrifugation, the supernatant was applied to a HisTrap (GE Healthcare) nickel column, and the bound protein was eluted with a linear imidazole gradient from 20 to 500 mm. And further purified by a HiLoad 16/60 Superdex 75 (GE Healthcare) gel filtration column. The pure protein were pooled and dialyzed in buffer (100 mm NaCl, 20 mm Tris, pH 7.5) with Slide‐A‐Lyzer (10000 MWCO). At last, the dialyzed protein were concentrated with to 5 mg mL^−1^ and stored in −80 °C freezer for future use.

In vitro MSH6_PWWP_ domain labeling with H3R2 (probe 1) and H3R2me2a (probe 2). H3R2 (probe 1, 10 µm) or H3R2me2a (probe 2, 10 µm) was incubated with MSH6_PWWP_ (2 µm) in the buffer containing 50 mm HEPES, 150 mm NaCl, 2 mm MgCl_2_, 10% glycerol, pH 7.5 for 30 min at 4 °C. The samples were irradiated at 365 nm for 20 min. 4 × Laemmli sample buffer was added (final diluted to 1 ×) and heated at 98 °C for 5 min. The samples were run on a 4–12% bis‐tris SDS‐PAGE gel and analyzed by western blot (detected by Streptavidin (HRP) anti‐biotin).

### Isothermal Titration Calorimetry Assay

Experiments were performed at 25 °C on a MicroCal PEAQ‐ITC automated (Malvern Instruments). Protein was loaded in the cell was titrated with 19 injections of peptides. The binding isotherm was fit with MicroCal PEAQ‐ITC Analysis software. The assay was done two times to confirm results.

### Generation of Diazirine Labeled Full‐Length Histone H3

Peptides SI‐1 or SI‐2 hydrazides (≈3 mg; 2 equiv.) were dissolved in degassed ligation buffer (6 m guanidinium chloride, 100 mm phosphate, pH 3.0) at −10 °C. 20 mm NaNO_2_ was added and generation of the corresponding acyl‐azide was left to proceed for 20 min at −10 °C. An equal volume of 200 mm (100 mm final concentration) of 4‐mercaptophenylacetic acid (MPAA) was added, and the pH was adjusted to 7.0. Thioester generation was left to proceed for 20 min at room temperature. The thioester was added directly to lyophilized recombinant H3_15‐135_^37^ (11.7 mg; 1 equiv.) and the reaction was incubated at rt for 4 h (the reaction was monitored to completion by LC‐MS). 100 mM TCEP was added for 20 min at rt to reduce co‐eluting MPAA disulfides, and the product was purified by semi‐preparative RP‐HPLC (gradient 10% −70% acetonitrile over 30 min). After lyophilization, ≈5 mg of full‐length diazirine labeled histone H3 products SI‐3 and SI‐4 was obtained.

### Octamer Assemble

Octamers were assembled as previously described with the following modifications.^[^ ^42^ ^]^ Recombinant and semisynthetic histones H2A, H2B, H4, and modified H3 (taking PTM, K‐dz, and biotin) were dissolved in histone unfolding buffer (20 mm Tris‐HCl, 6 m GdmHCl, 5 mm DTT, pH 7.5) and combined at equimolar ratios. The total histone concentration was adjusted to 1 mg mL^−1^. The mixture was placed in Slide‐A‐Lyzer MINI dialysis device (3500‐Da cutoff) and dialyzed at 4 °C against 3 × 600 mL of octamer refolding buffer (10 mm Tris‐HCl, 2 m NaCl, 1 mm EDTA, 1 mm DTT, pH 7.5) for at least 4 h for each step, with one dialysis step overnight. The mixture was then transferred to clean microcentrifuge tube and spun down at 17,000 g for 5 min at 4 °C to remove precipitate. The octamer in the supernatants was purified by HiLoad 26/60 Superdex 75 prep grade column to get pure H3R2 octamer (SI‐5) and H3R2me2a octamer (SI‐6), and concentrated to 25 µm for stock at −80 degree.

### Nucleosome Reconstitution

The protocol was described with the following modifications.^[^ ^42^ ^]^ 41 µg histone octamer (SI‐5 and SI‐6) was combined with 35 µg of 147 base pairs of 601 positioning sequence DNA (the molar ratio of octamer: DNA is 1:1) in 50 µL of octamer refolding buffer (10 mm Tris‐HCl, 2 m NaCl, 1 mm EDTA, 1 mm DTT, pH 7.5). The mixture was transferred to a SlideA‐Lyzer MINI dialysis device (3500‐Da cutoff) and dialyzed against 400 mL nucleosome reconstitution start buffer (10 mm Tris‐HCl, 2 m KCl, 1 mm EDTA, 1 mm DTT, pH 7.8). Subsequently, 2 L of nucleosome reconstitution buffer (10 mm Tris‐HCl, pH 7.5, 250 mm KCl, 1 mm EDTA, 1 mm DTT) was added to the extended solution at a rate of 1 mL mi^−1^n using a peristaltic pump, followed by final dialysis steps against nucleosome reconstitution buffer overnight. The dialysis mixture was transferred to a microcentrifuge tube, and any precipitate was removed by centrifugation to get nucleosome probe 9 and probe 10. The final nucleosomes were quantified by UV spectroscopy at 260 nm. The quality of the individual nucleosomes was assessed by polyacrylamide electrophoresis (5% acrylamide gel), followed by ethidium bromide staining and comassie blue staining. At last, the nucleosome was concentrated to 4 µm using centrifugal filters (30 KDa cutoff).

### In Vitro H3R2/H3R2me2a Nucleosome Pull‐Down

The protocol refers to general pull‐down assay by peptides with the following modifications. After incubating and crosslinking with 0.25 µµ nucleosome probes 9 and 10 separately, the protein samples were denatured in 0.5% SDS buffer by heating at 98 °C for 5 min. then the targeting protein was enriched with high‐capacity streptavidin agarose resin separately. The enriched protein on beads were released in 4 × Laemmli sample buffer by heating at 98 °C for 20 min. At last, the samples were run on a 4–12% bis‐tris SDS‐PAGE gel and analyzed by western blot. The assay was done two times to confirm results.

Generation of Npu^C^‐H3_15‐135_ plasmid expressing in mammalian cell. Npu^C^‐H3_15‐135_ was constructed using Gibson Assembly (New England Biolabs) following the manufacturer's instructions.

### Flag‐H3_1‐9_‐Npu^C^‐H3_15‐135_

GDYKDDDDKGARTKQTARKGIKIATRKYLGKQNVYDIGVERDHNFALKNGFIASNCFRKQLATKAARKSAPSTGGVKKPHRYRPGTVALREIRRYQKSTELLIRKLPFQRLVREIAQDFKTDLRFQSAAIGALQEASEAYLVGLFEDTNLCAIHAKRVTIMPKDIQLARRIRGERA

### Generation of H3_1‐14_‐Ava^N^ Protein

Purification of Ava^N^ refers to the described protocol.^[^ ^43^ ^]^ Native chemical ligation was used to generate H3_1‐14_‐Ava^N^ (probes 9 and 10). Peptides SI‐1 or SI‐2 hydrazides (≈1.8 mg; 4 equiv.) were dissolved in degassed ligation buffer (6 m guanidinium chloride, 100 mm phosphate, pH 3.0) at −10 °C in a salt‐ice bath. 20 mm NaNO_2_ was added and generation of the corresponding acyl‐azide was left to proceed for 20 min at −10 °C. An equal volume of 200 mm (100 mm final concentration) of 4‐mercaptophenylacetic acid (MPAA) was added, and the pH was adjusted to 7.0. Thioester generation was left to proceed for 20 min at room temperature. The thioester was added directly to lyophilized recombinant Ava^N^ (2.9 mg; 1 equiv.) and the reaction was incubated at rt for 4 h (the reaction was monitored to completion by LC‐MS). 100 mm TCEP was added for 20 min at rt to reduce co‐eluting MPAA disulfides, and the product was purified by semi‐preparative RP‐HPLC (gradient 10%−70% acetonitrile over 30 min). After lyophilization, ≈1.8 mg product was obtained. Lyophilized H3_1‐14_‐Ava^N^ was dissolved in PBS (pH 7.4) with 6 m urea and 1 mm DTT at 20–40 µm final concentration (≈1 mg mL^−1^). Stepwise dialysis was performed in PBS (pH 7.4) and 1 mm DTT with 4, 2, and 0 m urea for 2 h each, followed by 1×16 h additional dialysis step with 0 m urea. The refolded H3_1‐14_‐Ava^N^ was used to incubate with isolated nuclei.

### Protein Trans‐Splicing on Chromatin in the Isolated Nuclei

It refers to the protocol with the following modification.^[^ ^10^ ^]^ the frozen cell pellets 1×10^7^ HEK 293T cells expressing FLAG‐H3_1‐9_‐Npu^C^‐H3_15‐135_ were lysed by hypotonic lysis in 1 mL RSB buffer (10 mm tris, 15 mm NaCl, 1.5 mm MgCl_2_, Roche cOmplete EDTA‐free protease inhibitors, pH 7.6) for 10 min on ice. The crude nuclei were isolated by centrifugation at 400 g for 5 min at 4 °C. The nuclei were re‐suspended in 1 mL RSB buffer, and homogenized with ten strokes of a loose pestle dounce homogenizer, and pelleted at 400 g for 5 min at 4 °C. The nuclei were re‐suspended in 1 mL crosslinking buffer (20 mm HEPES, 1.5 mm MgCl_2_, 150 mm KCl, Roche cOmplete EDTA‐free protease inhibitors, pH 7.6) and centrifuged at 400 g for 5 min at 4 °C. Finally, the nuclei were re‐suspended in 300 µL of crosslinking buffer per 1×10^7^ cells. To the isolated nuclei was added H3R2‐Ava^N^ (probe 11) at the final concentration of 0.5 µm. After incubation at 37 °C for 30 min, the nuclei were washed with crosslinking buffer three times to remove excessive H3R2‐Ava^N,^ and the nulcei were lysed with 10 strokes of a tight pestle Dounce homogenizer in RSB buffer with 1% Triton x‐100. The nucleoplasm was separated by a 10 min centrifugation at 4 °C, 10,000 g, and the chromatin pellet was resuspended in SDS‐RIPA lysis buffer (25 mm Tris pH 8.0, 150 mm NaCl, 1% SDS, 1% NP‐40, 1% deoxycholate) and sonicated for 5 s at 35% amplitude. Then the samples were dissolved in 4 × Laemmli sample buffer by heating at 98 ℃C for 10 min. At last, the samples were run on a 4–12% bis‐tris SDS‐PAGE gel and analyzed by western blot. The assay was done two times to confirm results.

### Protein Trans‐Splicing Mediated H3R2/H3R2me2a Chromatin Pull‐Down

It refers to the described protocol with the following modification.^[^ ^10^ ^]^ The frozen 2×10^8^ HEK293T cells were used in the following steps. The extraction of intact nuclei was as same as the protocol for protein trans‐splicing on chromatin. The intact nuclei were incubated with 2 μm H3R2_(1‐14)_‐Ava^N^/ H3R2me2a_(1‐14)_‐Ava^N^ for 1 h at 37 °C, then the nuclei were washed with crosslinking buffer three times to remove excessive H3R2‐Ava^N^ and free histone H3 (not incorporated into chromatin) and they were placed on the ice and irradiated with 365 nm UV light for 30 min. 1 mm CaCl_2_ and micrococcal nuclease was added to digest the chromatin to mono‐ and di‐nucleosome (Note: without digestion by micrococcal nuclease, it was not efficient to release histones from chromatin in the following steps). Then the mono‐ and di‐nucleosome were denatured in 0.5% SDS buffer by heating at 98 °C for 5 min (To release histones from chromatin). Then the targeting protein attached to histone H3 (H3 fused with target protein via crosslinking) was pulled down with high‐capacity streptavidin agarose resin separately. The enriched protein on beads were released in 4 × laemmli sample buffer by heating at 98 °C for 20 min. At last, the samples were run on a 4–12% bis‐tris SDS‐PAGE gel and analyzed by western blot. The assay was done two times to confirm results.

### Homology Modeling and Flexible Peptide Docking of H3 Peptide and DPF2_PHD_ Domain

The initial model of DPF2 bound to H3 was first generated based on the crystal structure of DPF3‐H3 complex (PDB ID 5SZC) using SWISS‐MODEL (https://swissmodel.expasy.org).^[^ ^44^ ^]^ H3 peptide was then docked against DPF2 using Rosetta FlexPepDock,^[^ ^45^ ^]^ a high‐resolution peptide‐protein docking protocol implemented in the Rosetta framework. The structural figure was created using the PYMOL program.

### cBAF^WT^ and cBAF^MUT^ Complex Purification

cBAF^WT^ and cBAF^MUT^ complex were purified as described previously with several modifications.^[^ ^36^ ^]^ HEK293T cell lines stably expressing either HA‐tagged DPF2^WT^ or HA tagged DPF2^MUT^ (for cBAF) were grown on 15 cm dishes. Cell suspension was then centrifuged for 5 min at 4 °C at 4000 g. Cell pellets were resuspended in HB (10 mm Tris‐HCl, pH 7.5, 10 mm KCl, 1.5 mm MgCl2, 1 mm DTT, and 1 mm PMSF) and homogenized in Dounce homogenizers. The suspension was pelleted for 30 min at 4 °C at 4000 g and nuclear pellets were resuspended in pre‐extraction buffer (50 mm Tris‐HCl, pH 7.5, 100 mm KCl, 1 mm MgCl_2_, 1 mm EDTA, 1 mm, 0.1% (v/v) NP40, 1 mm DTT, 1 mm PMSF and protease inhibitor cocktail. After pelleting for 10 min at 4 °C at 4000 g, nuclei were resuspended in high‐salt buffer (HSB; 50 mm Tris‐HCl, pH 7.5, 300 mm KCl, 1 mm MgCl_2_, 1 mm EDTA, 1 mm, 1% NP40, 1 mm DTT, 1 mm PMSF and protease inhibitor cocktail) and incubated with rotation for 1 h at 4 °C. Homogenates were centrifuged at 20 000 rpm in a SW32Ti rotor (Beckman Coulter) for 1 h at 4 °C and the supernatant collected. Nuclear extracts were filtered with a 0.45 µm filter and rotated overnight at 4 °C with HA magnetic resin (Thermo Fisher Scientific). The beads were washed in HSB and proteins were eluted by incubating the beads four times for 1.5 h with 1 mg mL^−1^ of HA peptide in HSB. Eluates were than separated using differential centrifugation in 10–30% glycerol gradients. Fractions containing complexes of interest were collected, concentrated, and quantified with silver staining.

### Chromatin Remodeling Assay

EpiDyne‐FRET nucleosome (150 nm, Epicypher (16‐4201)) was incubated with cBAF^WT^ or cBAF^MUT^ (20 nm) chromatin remodeler in 20 µL buffer (20 mm HEPES, 4 mm Tris, pH 7.5, 60 mm KCl, 10 mm MgCl_2_, 10% glycerol). Upon the addition of 2 mm ATP, reactions were read at 0, 5, and 8 min by SpectraMax iD5 (excitation : 535 nm; Emission : 569 and 685 nm) and SoftMax Pro 7. Three biological replicates were conducted (N = 3).

## Conflict of Interest

The authors declare no conflict of interest.

## Supporting information

Supporting Information

Supporting Information

## Data Availability

The data that support the findings of this study are available in the supplementary material of this article.
