# Supplementary material for: Development of Complementary Photo‐arginine/lysine to Promote Discovery of Arg/Lys hPTMs Interactomes
Source: Adv Sci (Weinh). 2024 Jan 31;11(14):2307526. doi: 10.1002/advs.202307526 (PMC11005723; doi:10.1002/advs.202307526)
Supplement: Supplementary file 1 — Supporting Information [file ADVS-11-2307526-s002.pdf]

ADVANCED SCIENCE
Open Access

Supporting Information

for *Adv. Sci.*, DOI 10.1002/advs.202307526

Development of Complementary Photo-arginine/lysine to Promote Discovery of Arg/Lys hPTMs Interactomes

Yu Zong, Nicole Weiss, Ke Wang, Alexandra E. Pagano, Søren Heissel, Sumera Perveen and Jian Huang*

Supplementary Information

Development of Complementary Photo-arginine/lysine to Promote Discovery of Arg/Lys hPTMs Interactomes

Yu Zong[*]*, Nicole Weiss, Ke Wang, Alexandra E. Pagano, Søren Heissel, Sumera Perveen, Jian Huang*

Yu Zong, Ke Wang

Chemical Biology Program, Memorial Sloan Kettering Cancer Center, New York, USA

E-mail: zongy@mskcc.org

Nicole Weiss

Program of Pharmacology, Weill Cornell Medical College of Cornell University, New York, USA

Alexandra E. Pagano, Søren Heissel

Proteomics Resource Center, Rockefeller University, New York, USA

Sumera Perveen

Structural Genomics Consortium, University of Toronto, Toronto, Canada

Jian Huang

Department of Molecular Biology, Princeton University, Princeton, USA

Table of contents

Figure. S1 Reactivity of photo-arginine to six protected amino acids 3

Figure. S2 ITC data of H3R2 peptides and $UHRF1_{PHD}$ domain 3

Figure. S3 Representative MS spectrum for the peptides 4

Figure. S4 In vitro labeling of $MSH6_{PWWP}$ by probes **1** and **2**..4

Figure. S5 Comparing the δ-photo-lysine and photo-leucine probes labeling 4

Figure. S6 Generation of nucleosome probes **7** and **8**...5

Figure. S7 WB analysis of H3 trans-splicing on chromatin 5

Figure. S8 Silver stain of $cBAF^{WT}$ and $cBAF^{MUT}$ complex...6

Table. S1 Histone binding protein in SILAC dataset. 6

General materials 7

Chemical synthesis of R-dz and K-dz...7

NMR data...12

MS data 22

Reference...26

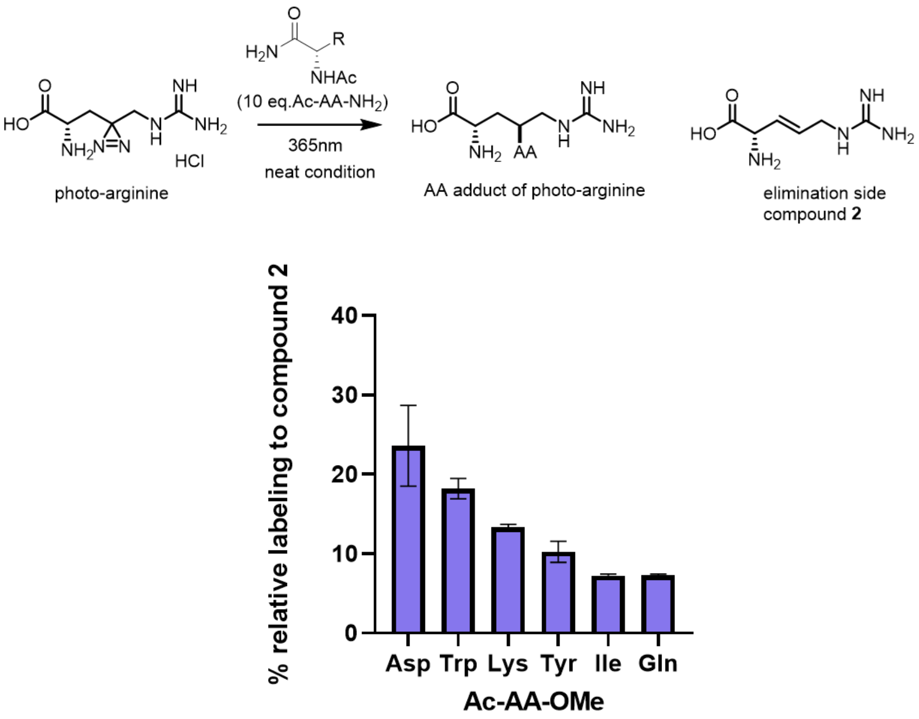

Figure S1. Reactivity of photo-arginine to six protected amino acids.

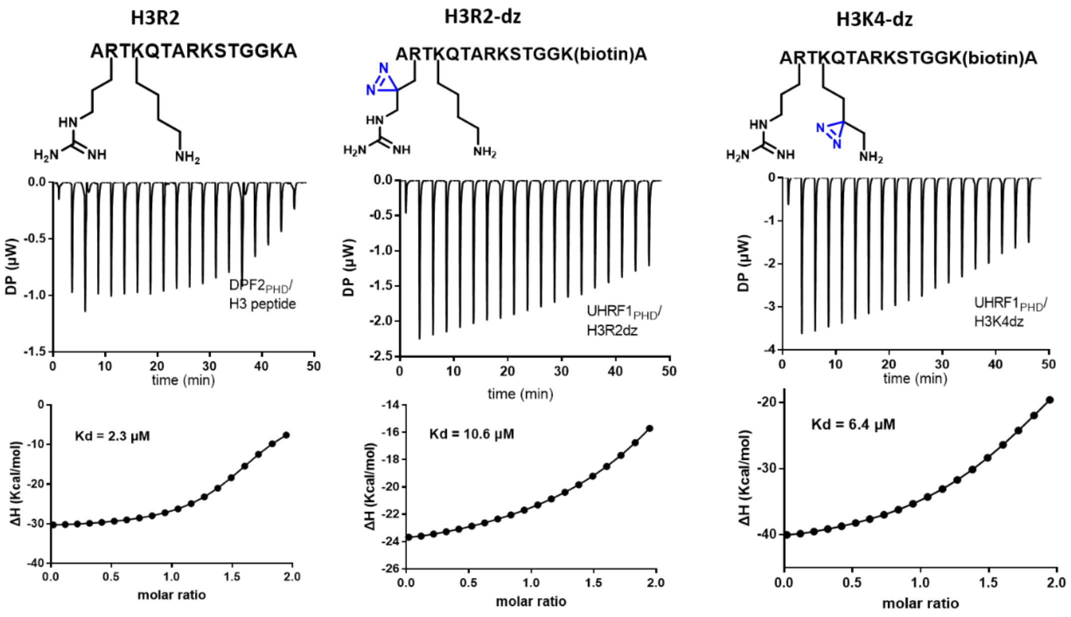

Figure S2. ITC data of H3R2/H3R2(dz)K14biotin/H3K4(dz)K14biotin and UHRF1$_{PHD}$ domain, n=2.

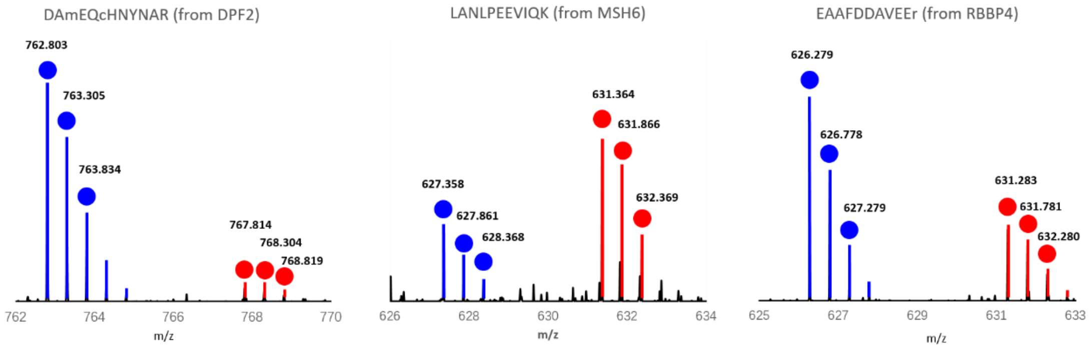

Figure S3. Representative MS spectrum for the peptides derived from DPF2, MSH6 and RBBP4 (Light peptides were from H3R2 group and heavy peptides were from H3R2me2a group).

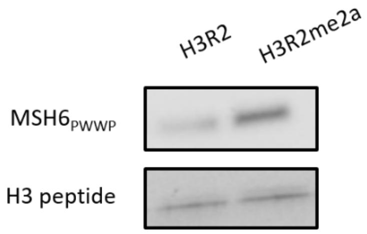

Figure S4. Labeling of $MSH6_{PWWP}$ (2 µM) by H3R2 and H3R2me2a probes (10 µM). n= 2, with representative data shown.

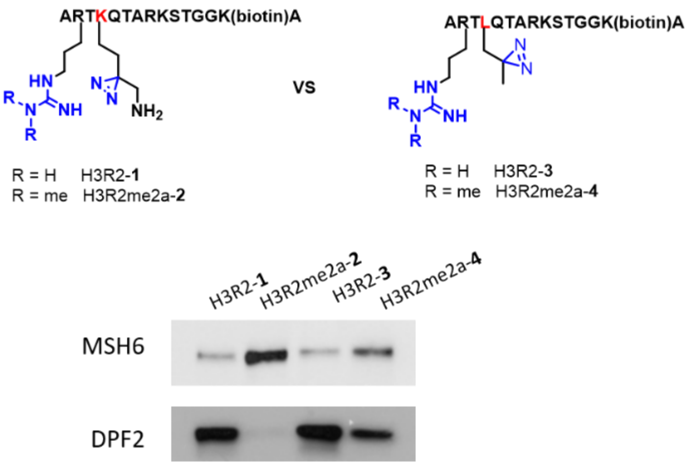

Figure S5. Comparing the δ-photo-lysine and photo-leucine probes labeling efficiency.

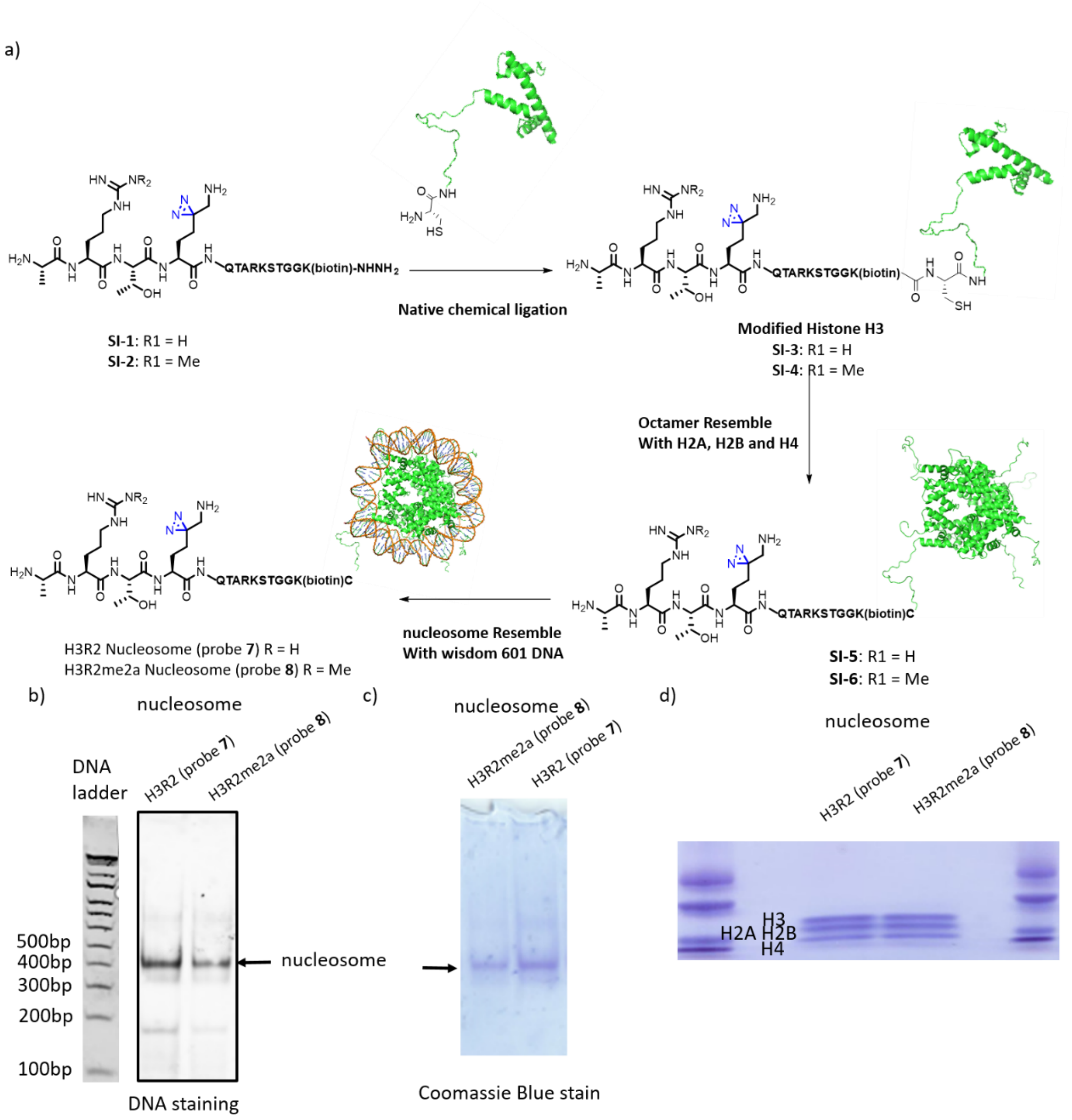

Figure S6. Process of generation of H3R2/H3R2me2a nucleosome. a) Schematic of generation of diazirine labeled H3R2/H3R2me2a nucleosome; b) Ethidium bromide staining of the nucleosome on the 5% acrylamide gel; c) Coomassie blue staining of nucleosome; d) Coomassie blue staining of H2A, H2B, H3 and H4 of the nucleosome.

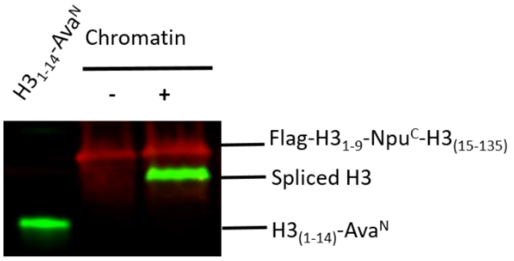

Figure S7. WB analysis of H3 trans-splicing on chromatin. n=2, with representative data shown.

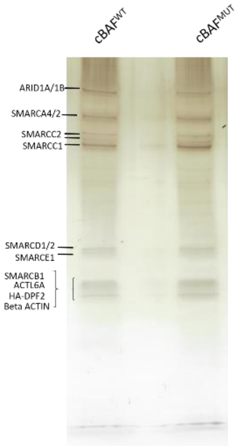

Figure S8. Silver stain of purified cBAF^{WT} and cBAF^{MUT} complex.

Table 1 Histone binding protein in SILAC dataset.

SILAC Dataset	Histone binding target
H3R2me2a/H3R2	MSH6, RCC1, ZZZ3, SMARCC2, SMARCC1, SMARCA4, MCM2, TP53BP1, CBX3, BPTF, ANP32E, PTMA, DEK, CHD4, ANP32A, NPM1, SET, CHD3, SSRP1, RBBP4, NOC2L, ZMYND8, BRD3, NASP, RBBP7, NAP1L1, NAP1L4, UHRF1, BRD4, BRD2, SETD1A, DPF2
H4K5me/ H4K5	SCML2, SMARCC2, PHF6, CBX1, DEK, TP53BP1, MBTD1, NASP, SMARCA4, TBL1X, HPF1, NAP1L4, ANP32E, IPO7, CHD4, VRK1, ANP32A, BRD7, UHRF1, DNAJC2, SET, TBL1XR1, SMARCA5, YEATS4, CBX3, RBBP7, L3MBTL2, CBX5, MCM2, NPM3, WDR5, GRWD1, NOC2L, HDAC2, PTMAP4, PTMA, NPM1, NAP1L1, RBBP4, CHD8, PHF10, SRCAP, RCC1, DNAJC9, SSRP1, DPF2, UIMC1, SMARCC1, ANP32B
H4K5me2/ H4K5	SCML2, SMARCC2, PHF6, CBX1, DEK, TP53BP1, MBTD1, NASP, SMARCA4, TBL1X, HPF1, NAP1L4, ANP32E, IPO7, CHD4, VRK1, ANP32A, BRD7, UHRF1, SAMD1, SET, TBL1XR1, SMARCA5, YEATS4, CBX3, RBBP7, L3MBTL2, CBX5, MCM2, NPM3, GRWD1, NOC2L, SMARCA2, HDAC2, PTMAP4, PTMA, NPM1, NAP1L1, RNF20, RBBP4, CHD8, SRCAP, RCC1, DNAJC9, SSRP1, DPF2, UIMC1, SMARCC1, ANP32B
H4K5acK8ac/ H4K5	BRD9, PHF6, DEK, RRP8, CHD4, BRD4, UHRF1, UHRF2, CBX5, MCM2, BPTF, HDAC2, PTMA, NPM1, LRWD1, NAP1L1, MYSM1, RNF20, SMARCA4, SSRP1, UIMC1, SMARCC1, CBX2, SCML2, SMARCC2, MORC3, TP53BP1, NASP, PHF8, HIRA, SMARCA2, VRK1, CBX8, ANP32A, ZZZ3, DNAJC2, HDGFL2, SET, TBL1XR1, SMARCA5, CBX3, RBBP7, KMT2D, ZMYND8, NPM3, BRD2, YEATS4, CXXC1, RBBP4, MSH6, CHD8, PHF10,

	KDM1B, RCC1, NAP1L4, RBBP5, DPF2, BRD3, TTLL12

General materials. Commonly used chemical reagents were purchased from Sigma Aldrich (St. Louis, MO) and Thermo Fisher (Waltham, MA), and used without further purification. All amino acid without modifications, Cl-TCP(Cl) ProTide and Rink amide ProTide resin were ordered from CEM. All modified amino acids (Fmoc-Lys(Ac)-OH, Fmoc-Lys(Biotin)-OH, Fmoc-Lys(Boc, Me)-OH, Fmoc-Lys(Me2)-OH·HCl, Fmoc-Arg(me2, Pbf)-OH (asymmetrical)) were from Sigma Aldrich. N-Boc-allylglycine was purchased from aablocks. Anti-MSH6 (mAb 12988S, cell signaling technology); Anti-Spin1 (12105-1-AP, Proteintech); Anti-ING2 (11560-1-AP, Proteintech); WDR5 (13105S, cell signaling technology); Anti-DPF2 (sc-514297, Santa Cruz Biotechnology); Anti-RBBP4/7 (9067S, Cell signaling technology); Anti-SETD1A (61702S, Cell signaling technology); Anti-L3MBTL2 (sc-365134, Santa Cruz Biotechnology); Anti-YEATS4 (sc-393708, Santa Cruz Biotechnology); Anti-Rabbit (926-68071, LI-COR); Anti-Mouse (926-68070, LI-COR); Anti-biotin (926-32230, LI-COR); Streptavidin (HRP) (ab7403, ABCAM); Anti-Rabbit IgG (7074S, Cell signaling technology); Anti-Mouse IgG (31430, Thermo Fisher Scientific). His-$DPF2_{PHD}$ domain in pET28-MHL vector (addgene, 25222); $MSH6_{PWWP}$ domain in pET28-MHL vector (addgene, 162261); RBBP4 and RBBP7 in pFBOH-LIC vector; Flag-$H3_{1\text{-}9}$-Ava^{N}-$H3_{15\text{-}135}$ in pEGFP-N1 vector (EPF was replaced by Flag-$H3_{1\text{-}9}$-Ava^{N}-$H3_{15\text{-}135}$). Nucleosome Remodeling Assay Substrate (16-4201, Epicypher). Gibson Assembly Master Mix (M5510A, New England biolabs inc.). Q5 Site-directed mutagenesis kit (E0554S, New England biolabs inc.).

Chemical synthesis of R-dz and K-dz

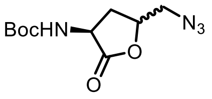

d*r*($c_{is/trans}$) =7:1
2

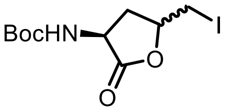

1-1

Synthesis of compound **2**. Compound **2** was synthesized by reported method with the following modification[1]. *N*-Boc-allylglycine (6.45 g, 30 mmol) was dissolved in 100 mL THF at 0 °C. Into this solution were added I_2 (22.9 g, 90 mmol), followed by $NaHCO_3$ (10.1 g, 120 mmol) dissolved in 100 mL H_2O. This reaction mixture was stirred at ambient temperature (30 °C) for 5 h and then diluted with 200 mL of EtOAc. This reaction was then quenched with 30 mL of saturated $Na_2S_2O_3$ solution. Sodium chloride was added to generate the saturated brine. The organic layer was washed three times with saturated brine and dried with anhydrous Na_2SO_4. The solvent was evaporated under vacuum to get the resulting crude intermediate compound **1-1** without purification. Compound **1-1** was then dissolved in 100 mL of DMF, followed by addition of NaN_3 (5.85 g, 90 mmol). The reaction mixture was stirred at 40 °C for 16 h and then diluted with 300 mL EtOAc. The organic layer was washed sequentially with water and saturated brine for three times, and dried with anhydrous Na_2SO_4. The solvent was evaporated under vaccum. The resulting crude product was purified by silica gel column chromatography (1:9 EtOAc/hexane from 1:9 to 1:1) to afford the desired product **2** with the overall yield of 85% (6.53 g). LRMS (ESI) calculated for $C_{10}H_{17}N_4O_4$ [M + H]$^+$ 257.13, found 257.33.

^{1}H NMR (600 MHz, $CDCl_3$): 5.14 (bs, 1H), 4.60-4.50 (m, 1H), 4.48-4.43 (m, 1H), 3.63 (dd, J = 13.5 Hz, 3.54 Hz 1H), 3.50 (dd, J = 13.5 Hz, 5.30 Hz, 1H), 2.79-2.74 (m, 1H), 2.04-1.99 (m, 1H), 1.45 (s, 9H); ^{13}C NMR (150 MHz, $CDCl_3$): 173.9, 155.3, 80.8, 75.7, 53.3, 51.0, 33.0, 28.3.

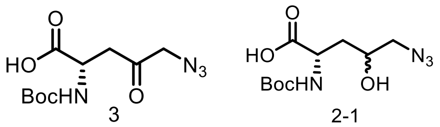

3 2-1

Synthesis of **3**. Compound **2** (3.7 g, 14.4 mmol) was dissolved in 120 mL THF/H_2O (v/v 3:1). Into the solution was added LiOH (1.03 g, 43 mmol). The reaction mixture was stirred at ambient temperature (23 °C) for 15 min. The resulting mixture was acidified with 10 % citric acid to pH 3.0 and diluted with 100 ml EtOAc. Sodium chloride was added to generate the saturated brine. The combined organic phase was dried with anhydrous Na_2SO_4. And concentrated under vacuum to give the crude compound **2-1** without further purification. The crude compound **2-1** was dissolved in 100 mL DCM. Into this solution was added of Dess–Martin periodinane (DMP) reagent (7.78 g, 16 mmol). This reaction mixture was stirred at ambient temperature (23 °C) for 30 min. The excessive DMP was consumed by 1 mL MeOH and the solvent was concentrated to 30 mL and filtrate the precipitation. The resulting crude product was purified by silica gel column chromatography with hexane: ethyl acetate /(100% to 25%) to afford the desired product **3** with the overall yield of 76% (3 g). HRMS (ESI) calculated for $C_{10}H_{16}N_4NaO_5$ $[M + Na]^+$ 295.1018, found 295.1024. ^{1}H NMR (600 MHz, CD_3OD): 4.54-4.50 (m, 1H), 4.12 (d, J = 18.5 Hz, 1H), 4.05 (d, J = 16.7 Hz, 1H), 3.00 (dd, J = 5.28 Hz, 17.0 Hz, 1H), 2.85 (dd, J = 6.84 Hz, 17.0 Hz, 1H), 1.44 (s, 9H). ^{13}C NMR (150 MHz, CD_3OD): 203.9, 174.9, 157.9, 80.9, 58.5, 50.8, 42.5, 28.8.

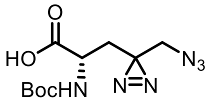

4

Synthesis of Compound **4**. Compound **3** (3.0 g, 11 mmol) was dissolved with 30 mL of 7 N NH_3 in MeOH. This solution was stirred at 0 °C for 1 h. Into this solution was added hydroxylamine-*O*-sulfonic acid (1.62 g, 14.3 mmol) dissolved in 5 mL of anhydrous methanol. This reaction mixture was then stirred at ambient temperature (23 °C) for 6 h. After filtering out the white precipitate, 7 N NH_3 in MeOH was evaporated. Then the reaction was subject to an ice bath, followed by addition of 30 mL of methanol and trimethylamine (2.78 mL, 20 mmol). Iodine (5.08 g, 20 mmol) was added batch by batch until the color of iodine persists. After 30 min, the reaction was quenched with saturated $Na_2S_2O_3$ solution. After the solvent was evaperated under vacuum, the reaction mixture was dissolved in 100 mL EtOAc, washed with saturated brine, and dried with anhydrous Na_2SO_4 and concentrated under vacuum. The resulting crude product was purified by silica gel column chromatography with 10:1 (v/v) DCM:MeOH to afford the desired Compound **4** with the yield of 27 % (0. 84 g). HRMS (ESI) calculated for $C_{10}H_{15}N_6O_4$ $[M - H]^-$ 283.1155. Found 283.1155. ^{1}H NMR (600 MHz, CD_3OD): 4.01 (dd, J = 10.20 Hz, J = 4.26 Hz, 1H), 3.38 (d, J = 14.16 Hz, 1H), 3.30 (d, J = 14.16 Hz, 1H), 2.10-2.06 (m, 1H), 1.75-1.70 (m, 1H), 1.47 (s, 9H). ^{13}C-NMR (150 MHz, CD_3OD): 174.8, 158.0, 80.9, 54.9, 50.7, 34.7, 28.9, 27.8.

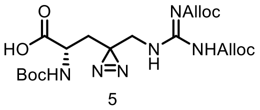

5

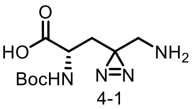

4-1

Synthesis of Compound **5**. Compound **4** (71 mg, 0.25 mmol) was dissolved in 10 mL of 20:1 (v/v) THF/H_2O, followed by addition of 1 M trimethylphosphine in THF (2.5 mL, 2.5 mmol) under a nitrogen atmosphere in dark. This reaction mixture was stirred at ambient temperature for 20 min, the solvent was evaporated to yield the crude Compound **4-1** without further purification. Compound **4-1** was dissolved in 5 mL dioxane/H_2O (5:2), and then mixed with Et_3N (0.21 mL, 1.5 mmol) and 1H-pyrazole-*N*,*N*'-di-alloc-1-carboxamidine[2] (0.21 g, 0.75 mmol). This reaction mixture was stirred at 40 ^oC for 2 h. The resulting solution was diluted with 20 mL of ethyl acetate and washed with saturated brine. The organic layer was dried with anhydrous Na_2SO_4 and evaporated in vacuo. The residue was purified by silica gel column chromatography with EtOAc/Hexane (from 1:4 to 4:1) to afford compound **5** with the yield of 75% (88 mg). HRMS (ESI) calculated for $C_{19}H_{28}N_6NaO_8$ $[M + Na]^+$ 491.1866. found 491.1855.^1H NMR (600 MHz, DMSO-d6): 11.47 (s, 1H), 8.32 (s, 1H), 7.60 (s, 1H), 5.97-5.92 (m, 2H), 5.38-5.27 (m, 3H), 5.18 (d, *J* =9.04, Hz, 1H), 4.67 (d, *J* =5.4 Hz, 2H), 4.51 (d, J =5.3 Hz, 2H), 3.56 (m, 1H), 3.43 (d, *J* =4.26, 2H), 1.87-1.83 (m, 1H), 1.70-1.68 (m, 1H), 1.39(s, 9H), ^{13}C NMR (150 MHz, DMSO-d6): 162.6, 155.2, 155.1, 133.4, 131.8, 118.9, 117.4, 104.2, 77.9, 66.5, 65.6, 42.5, 28.3, 27.4.

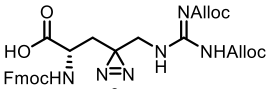

6

Synthesis of Compounds **6**. Compound **5** (68 mg, 0.15 mmol) was dissolved in 2 mL mixture solvent (TFA:DCM/1:4), these mixtures were stirred at 30 ^oC for 0.5 h. solvent was then completely removed in vacuo. The residues were dissolved with 2 mL saturated $NaHCO_3$ with the pH controlled in the range of 8~9. Into this solution was added with Fmoc-OSu (98 mg, 0.29 mmol) dissolved in 5 mL dioxane. The resulting reaction mixture was stirred at 30 °C overnight. After removing the dioxane under vacuum, the crude product was extracted with 10 mL EtOAc. The organic layers were washed with 1M HCl (aq.) and saturated brine and dried with anhydrous Na_2SO_4. After removing the solvent under vacuum, the residue was subject to silica gel column chromatography with 1:9 (v/v) DCM/MeOH to afford the desired products **6**. Then the desired product **6** was purified with 19×150mm column (XBridge Pre C18 5μm OBD) (10-95% acetonitrile with 0.1% formic acid 25min), followed by lyophilization to get final compound **6** (48mg, 56%).

HRMS (ESI) calculated for $C_{29}H_{31}N_6O_8$ $[M + H]^+$ 591.2203, found 591.2206. ^1H NMR (500 MHz, $CDCl_3$+5% D_2O) 7.74 (d, *J* = 7,5 Hz, 2H), 7.64-7.60 (m, 2H), 7.40-7.36 (m, 2H), 7.31-7.27 (m, 2H), 5.94-5.82 (m, 2H), 5.38-5.27 (m, 3H), 5.18 (d, *J* = 10.4 Hz, 1H), 4.64 (d, *J* = 5.9 Hz, 2H), 4.58 (dd, J = 13.3 Hz, 5.6 Hz, 1H), 4.55-4.38 (m, 4H), 4.24 (t, *J* = 7.0 Hz, 1H), 3.63 (d, *J* = 15.3 Hz, 1H), 3.22 (d, *J* = 15.3 Hz, 1H), 1.97 (dd, J = 15.1 Hz, 8.9 Hz, 1H), 1.90 (dd, J = 15.1 Hz, 4.1 Hz, 1H) ^{13}C NMR (150 MHz, $CDCl_3$+5% D_2O) 173.2, 163.0, 156.6, 156.0, 156.4, 153.5, 144.0, 143.9, 141.5, 132.6, 130.9, 127.8, 127.2, 125.3, 120.1, 120.0, 118.2, 67.5, 67.3, 66.5, 50.4, 47.3, 43.2, 34.1, 26.4.

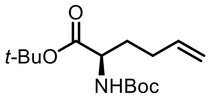

8

Synthesis of Compound **8**. Into 50 mL DCM in a 250 mL round bottom flask was dissolved compound **7** (two steps from commercial available starting material)[3] (7.0 g, 30.6 mmol). To this solution added *tert*-butyl 2,2,2-trichloroacetamidate (6.8 mL, 45 mmol) dissolved in 50 mL cyclohexane. Into this mixture was then added BF_3•Et_2O (0.37 mL, 3 mmol). After stirring this reaction at ambient temperature for 2 h, the resulting mixture was cooled in an ice bath, followed by addition of $NaHCO_3$ powder (840 mg, 10 mmol). This mixture was stirred at ambient temperature for 10 min and filtered. The filtrate was evaporated in vaccum and the residue was purified by silica gel column chromatography with 15:85 (v/v) EtOAc/Hexanes to afford the desired Compound **8** with the yield of 78% (6.83 g). ^{1}H NMR (500 MHz, $CDCl_3$) 5.81-5.74 (m, 1H), 5.05-4.95 (m, 3H), 4.18-4.16 (m, 1H), 2.11-2.05 (m, 2H), 1.90-1.70 (m, 2H), 1.45 (s, 9H), 1.42 (s, 9H). ^{13}C NMR (125 MHz, $CDCl_3$) 172.1, 155.5, 137.5, 115.6, 82.0, 80.0, 53.8, 32.5, 29.6, 28.5, 28.2. HRMS (ESI) calculated for $C_{15}H_{27}NNaO_4$ $[M + Na]^+$ 308.1838, found 308.1838.

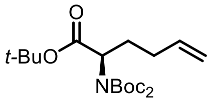

9

Synthesis of Compound **9**. To a solution of compound **8** (24 mmol, 6.83 g) in anhydrous acetonitrile (100 mL) was added DMAP (2.93 g, 24 mmol) followed by Boc_2O (27.5 mL, 120 mmol), and the mixture was refluxed 3 hours. TLC showed that starting material was still present; therefore, another portion of Boc_2O was added, and the mixture was refluxed for another 3 hours. The solvent was removed under reduced pressure, and the crude material was purified by flash chromatography on silica (hexanes/ethyl acetate 9:1) to give the desired product **9** (8.04 g, 87%). HRMS (ESI) calculated for $C_{20}H_{35}NNaO_6$ $[M + Na]^+$ 408.2362, found 408.2365. ^{1}H NMR (500 MHz, $CDCl_3$) 5.81-5.74 (m, 1H), 5.06-4.95 (m, 2H), 4.74-4.70 (m, 1H), 2.20-1.92 (m, 4H), 1.49 (s, 18H), 1.44 (s, 9H). ^{13}C NMR (125 MHz, $CDCl_3$) 170.1, 152.6, 137.8, 115.5, 82.9, 81.3, 58.5, 30.7, 28.9, 28.2.

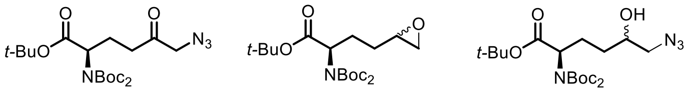

10 9-1 9-2

Synthesis of Compound **10**. To a solution of compound **9** (20.8 mmol, 8 g) in DCM (100 mL) was added mCPBA (5.38 g, 31.2 mmol), After stirring this reaction at ambient temperature (22 °C) for 3 h, The resulting solution was washed with saturated $NaHCO_3$(aq.) three times. The organic layer was dried with anhydrous Na_2SO_4 and evaporated in vacuo to get intermediate **10-1** without any purification for the next step. To a solution of compound **9-1** in DMF (100 mL) was added NH_4Cl (1.12 g, 21 mmol) and NaN_3 (10.9 g, 168 mmol). After stirring this reaction at 60 °C overnight, The resulting solution was diluted with ethyl acetate and was washed with water, saturated $NaHCO_3$ (aq.) and brine three times. The organic layer was dried with anhydrous Na_2SO_4 and evaporated in vacuo to get intermediate **9-2** without any purification for the next step. To a solution of compound **10-2** in DCM (100 mL) was

added Dess–Martin periodinane (DMP) reagent (8.5 g, 20 mmol), After stirring this reaction at 30 °C for 3 h, the resulting solution was washed with saturated $NaHCO_3$ (aq.) three times. The organic layer was dried with anhydrous Na_2SO_4 and evaporated in vacuo. And the crude material was purified by flash chromatography on silica (hexanes/ethyl acetate 4:1) to give the desired product **10** with the yield of 68 % (6.3 g) over three steps. HRMS (ESI) calculated for $C_{20}H_{34}N_4NaO_7$ $[M + Na]^+$ 465.2325, found 465.2315. ^1H NMR (500 MHz, $CDCl_3$) 4.70-4.65 (m, 1H), 3.92 (s, 2H), 2.57-2.43 (m, 3H), 2.15-2.10 (m, 1H), 1.50 (s, 18H), 1.44 (s, 9H). ^{13}C NMR (125 MHz, $CDCl_3$) 203.6, 169.3, 152.7, 83.4, 81.7, 58.2, 57.5, 36.8, 28.2, 28.1, 23.1.

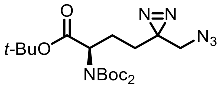

11

Synthesis of Compound **11**. Compound **10** (1.6 g, 3.6 mmol) was dissolved with 30 mL of 7 N NH_3 in MeOH. This solution was stirred at 0 °C for 1 h. Into this solution was added hydroxylamine-*O*-sulfonic acid (0.52 g, 4.6 mmol) dissolved in 3 mL of anhydrous methanol. This reaction mixture was then stirred at 30 °C for 6 h. After filtering out the white precipitate, 7 N NH_3 in MeOH was evaporated. Then the reaction was subject to an ice bath, followed by addition of 30 mL of methanol and trimethylamine (1mL, 7.2 mmol). Iodine (1.83 g, 7.2 mmol) was added batch by batch until the color of iodine persists. After 30 min, the reaction was quenched with saturated $Na_2S_2O_3$ solution. After the solvent was evaperated under vacuum, the reaction mixture was dissolved in 100 mL EtOAc, washed with s washed sequentially with saturated brine once, and dried with anhydrous Na_2SO_4 and concentrated under vacuum. The resulting crude product was purified by silica gel column chromatography with (hexanes/ethyl acetate 4:1) to afford the desired Compound **11** (245 mg, 15%). ^1H NMR (500 MHz, $CDCl_3$) 4.63-4.59 (m, 1H), 3.21 (d, J = 13.9, 1H), 3.13 (d, J = 13.9 Hz, 1H), 1.93-1.90 (m, 1H), 1.70-1.55 (m, 2H), 1.49 (s, 18H), 1.42 (s, 9H). ^{13}C NMR (125 MHz, $CDCl_3$) 169.3, 152.6, 83.3, 81.8, 58.2, 54.4, 36.8, 28.2, 28.1, 23.16. HRMS (ESI) calculated for $C_{20}H_{34}N_6NaO_6$ $[M + Na]^+$ 477.2438, found 477.2446.

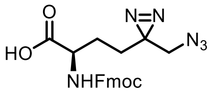

12

Synthesis of Compounds **12**. Compound **11** (45 mg, 0.1 mmol) was dissolved in 2 ml mixture solvent (TFA:DCM/95:5), these mixtures were stirred at 30 ^oC for 1 h. solvent was then completely removed in vacuo. The residues were dissolved with 2 ml saturated $NaHCO_3$ with the pH controlled in the range of 8~9. Into this solution was added with Fmoc-OSu (51 mg, 0.15 mmol) dissolved in 5 mL dioxane. The resulting reaction mixture was stirred at 30 °C overnight. After removing the dioxane under vacuum, the crude product was extracted with 10 mL EtOAc. The organic layers were washed with 1M HCl (aq.) and saturated brine and dried with anhydrous Na_2SO_4. After removing the solvent under vacuum, the residue was subject to silica gel column chromatography with 1:9 (v/v) DCM/MeOH to afford the desired product **13**. Then the desired product **12** was purified with 19×150mm column (XBridge Pre C18 5μm OBD) (10-95% acetonitrile with 0.1% formic acid 25min), followed by lyophilization to get final compound **12** (25 mg, 55%). HRMS (ESI) calculated for

$C_{21}H_{20}N_6NaO_4$ $[M + Na]^+$ 443.1444, found 443.1437. ^1H NMR (500 MHz, DMSO-d6+ 5% CD3OD) 7.88 (d, *J* = 7,5 Hz, 2H), 7.70 (d, *J* = 7,3 Hz, 2H), 7.43-7.33 (t, *J* = 7,4 Hz, 2H), 7.34-7.30 (m, 2H), 4.32-4.21 (m, 3H), 3.91-3.89 (m, 1H), 3.43 (d, *J* = 2.3 Hz, 2H), 1.56-1.42 (m, 4H). ^{13}C NMR (DMSO-d6+ 5% CD3OD) 173.3, 156.2, 143.9, 140.8, 127.7, 127.1, 125.3, 120.2, 65.7, 53.0, 52.9, 46.7, 28.2, 27.3, 24.9.

NMR data

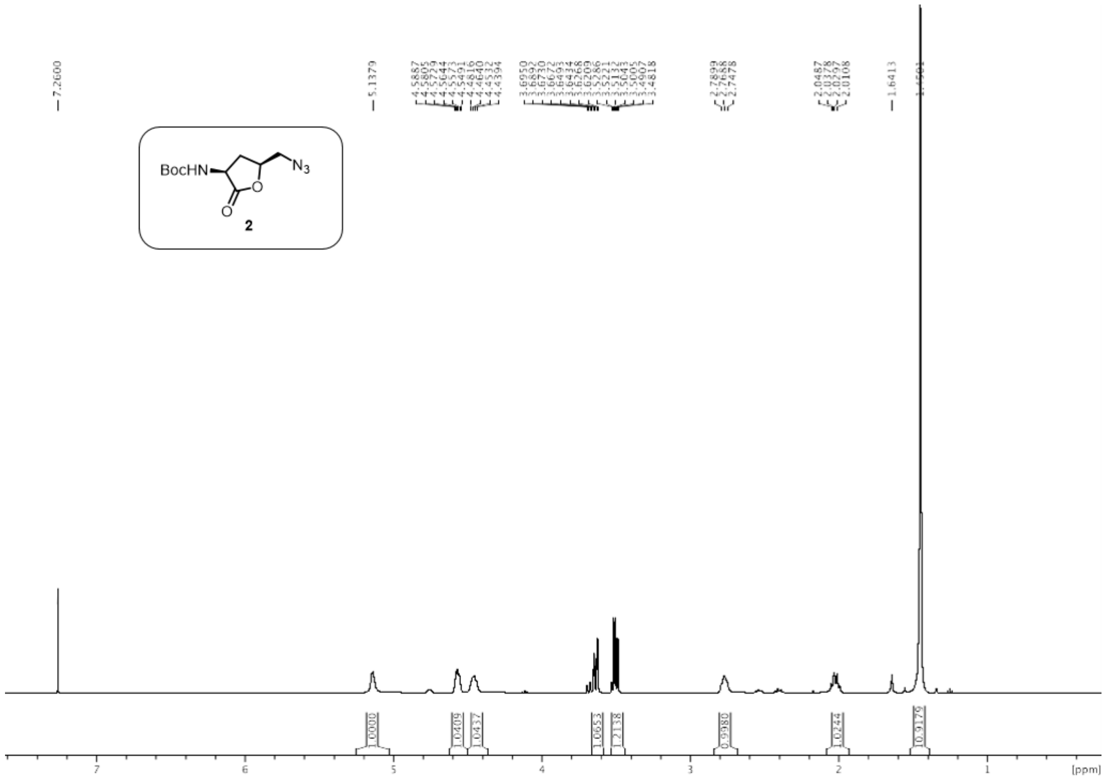

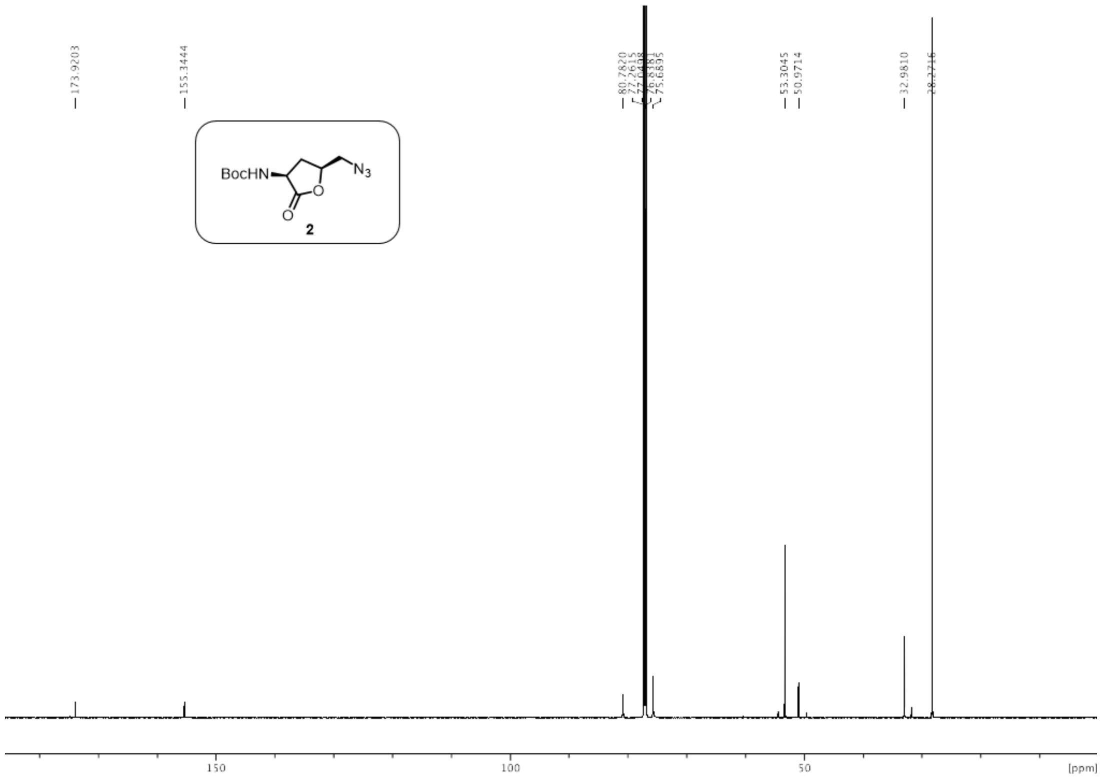

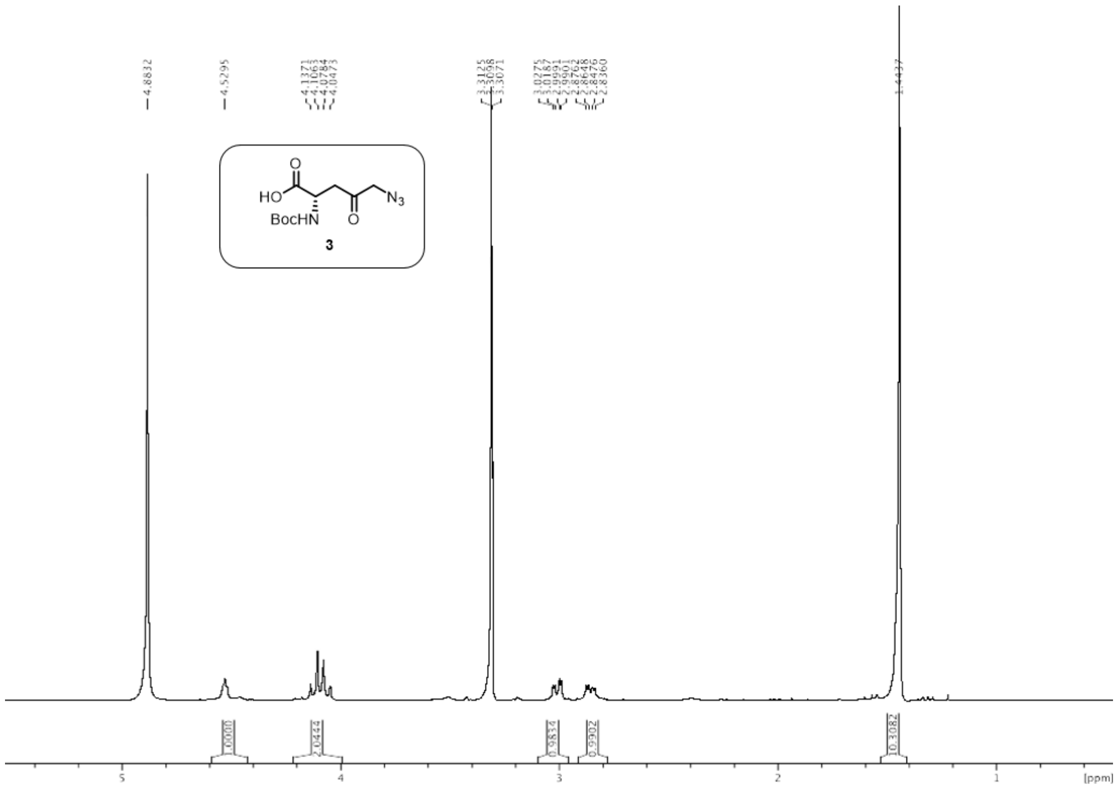

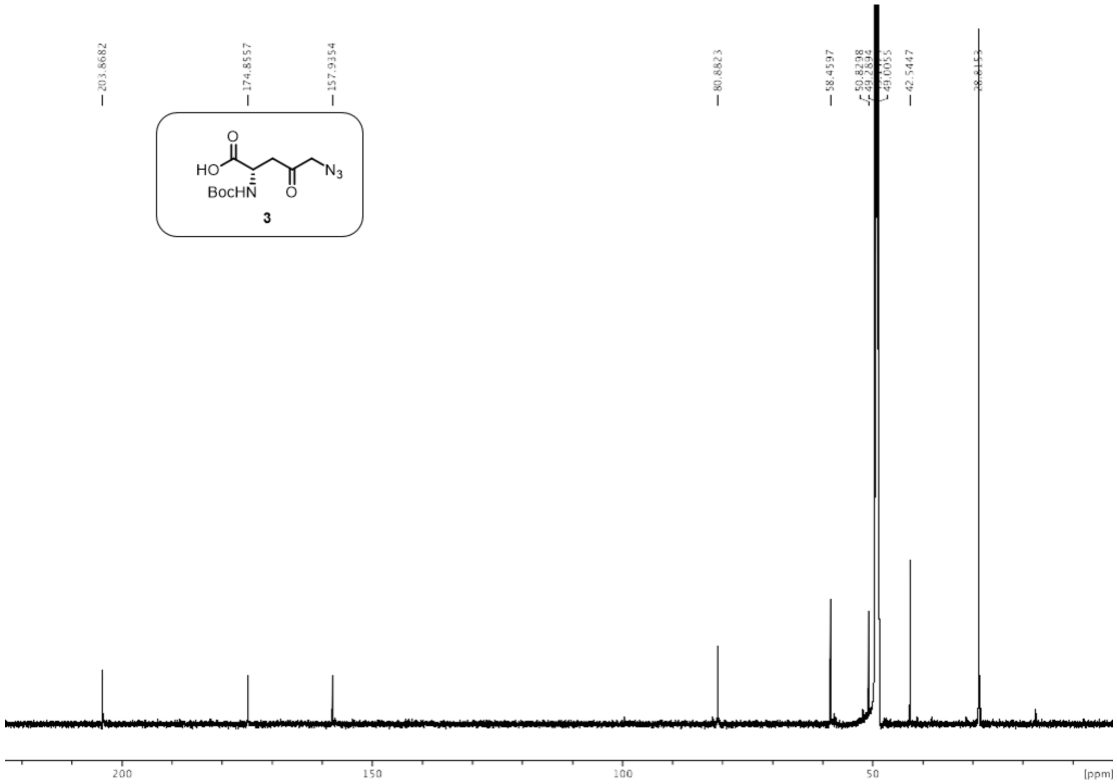
203.8682
174.8557
157.9354
80.8823
58.4597
50.8298
49.2894
49.0055
42.5447
28.8153
HO
O
N3
BocHN
O
3
200
150
100
50
[ppm]

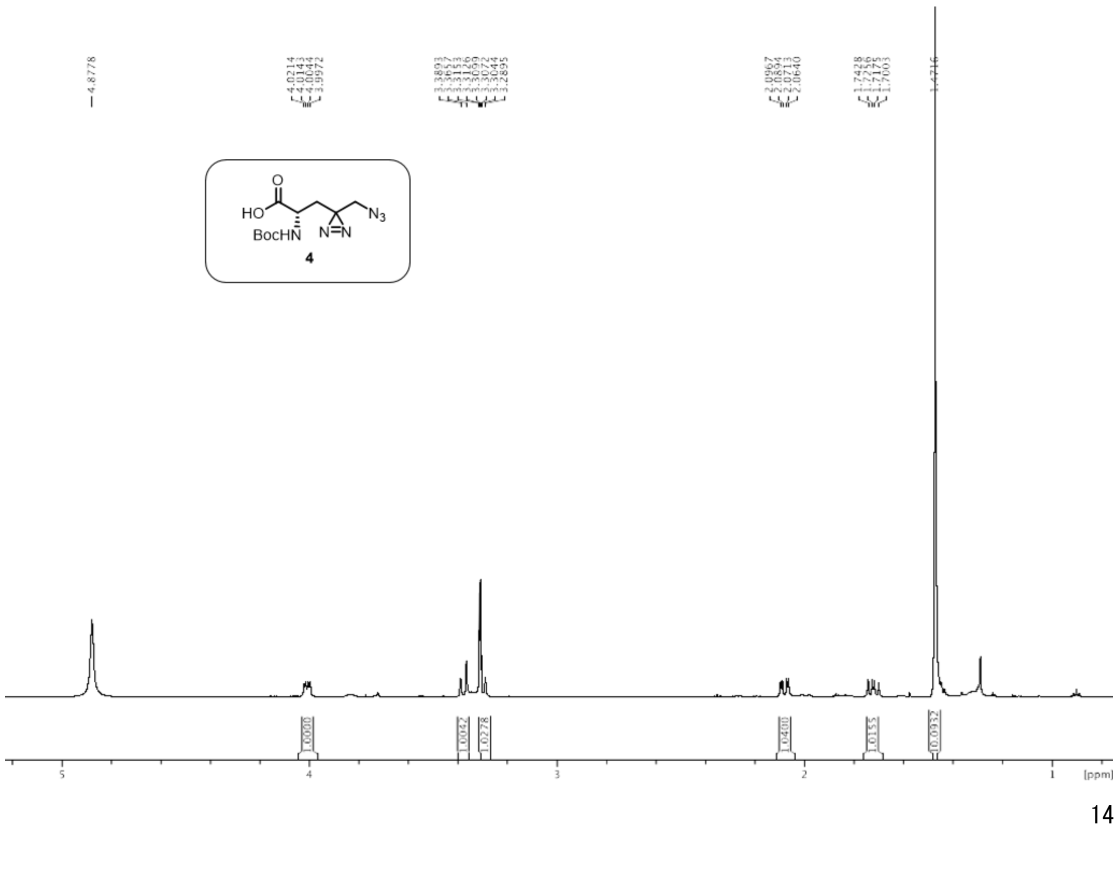
4.8778
4.0214
4.0143
4.0044
3.9972
3.3893
3.3657
3.3153
3.3126
3.3099
3.3072
3.3044
3.2895
2.0967
2.0894
2.0713
2.0640
1.7428
1.7256
1.7175
1.7003
1.4716
HO
O
N3
BocHN
N=N
4
1.0000
1.0042
1.0278
1.0400
1.0155
10.0932
5
4
3
2
1
[ppm]

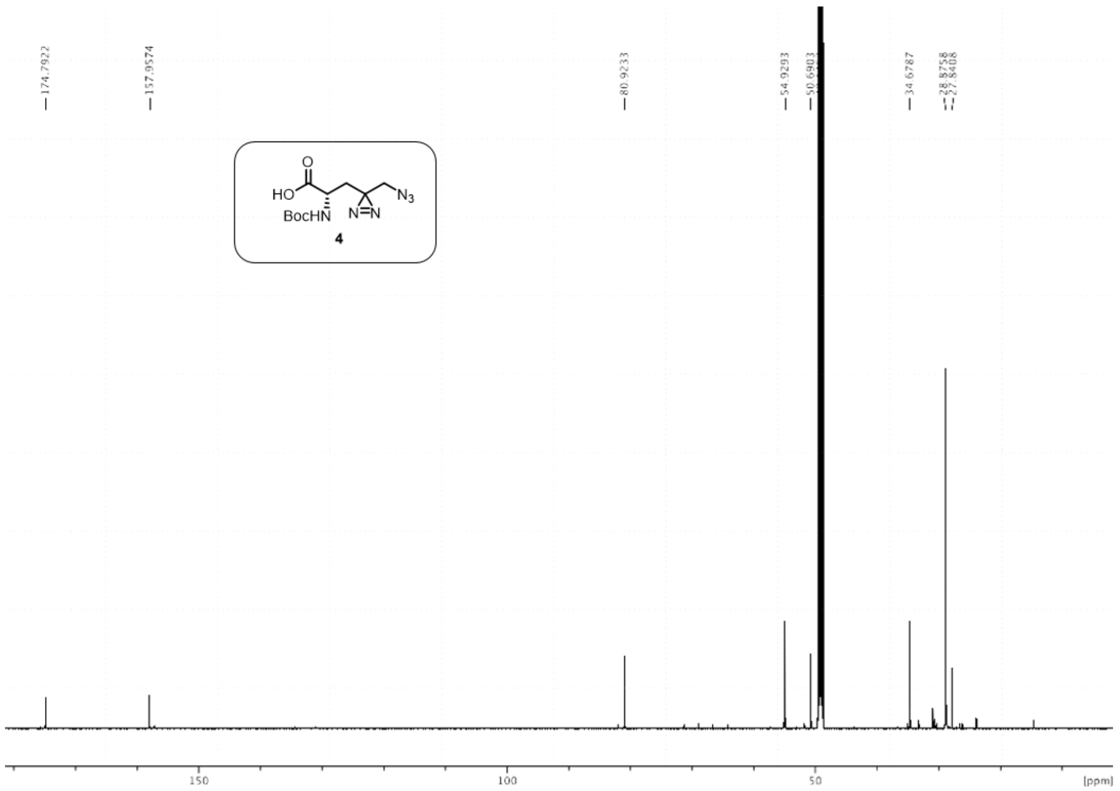

174.7922
157.9574
80.9233
54.9293
50.6903
34.6787
28.8758
27.8408
HO
O
BocHN
N=N
N_3
4
150
100
50
[ppm]

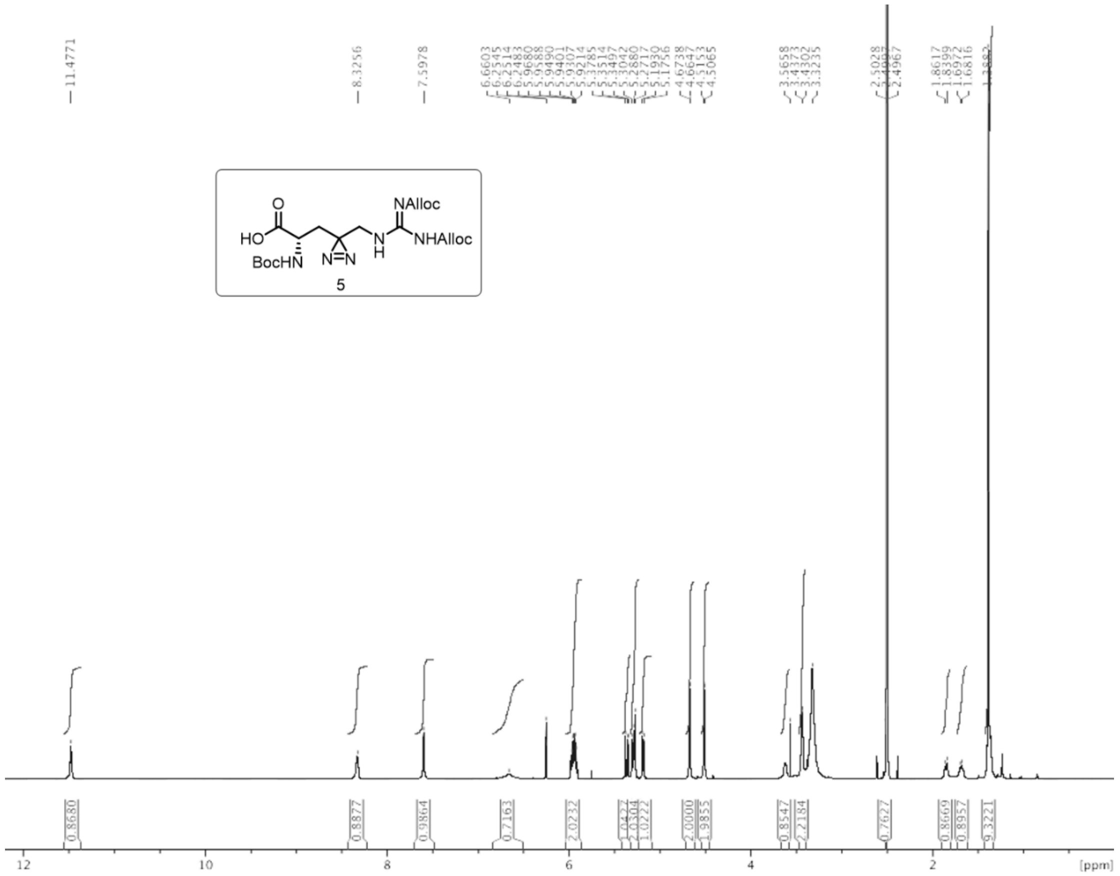
NAlloc
HO
NHAlloc
BocHN
N=N
H
5
12
10
8
6
4
2
[ppm]

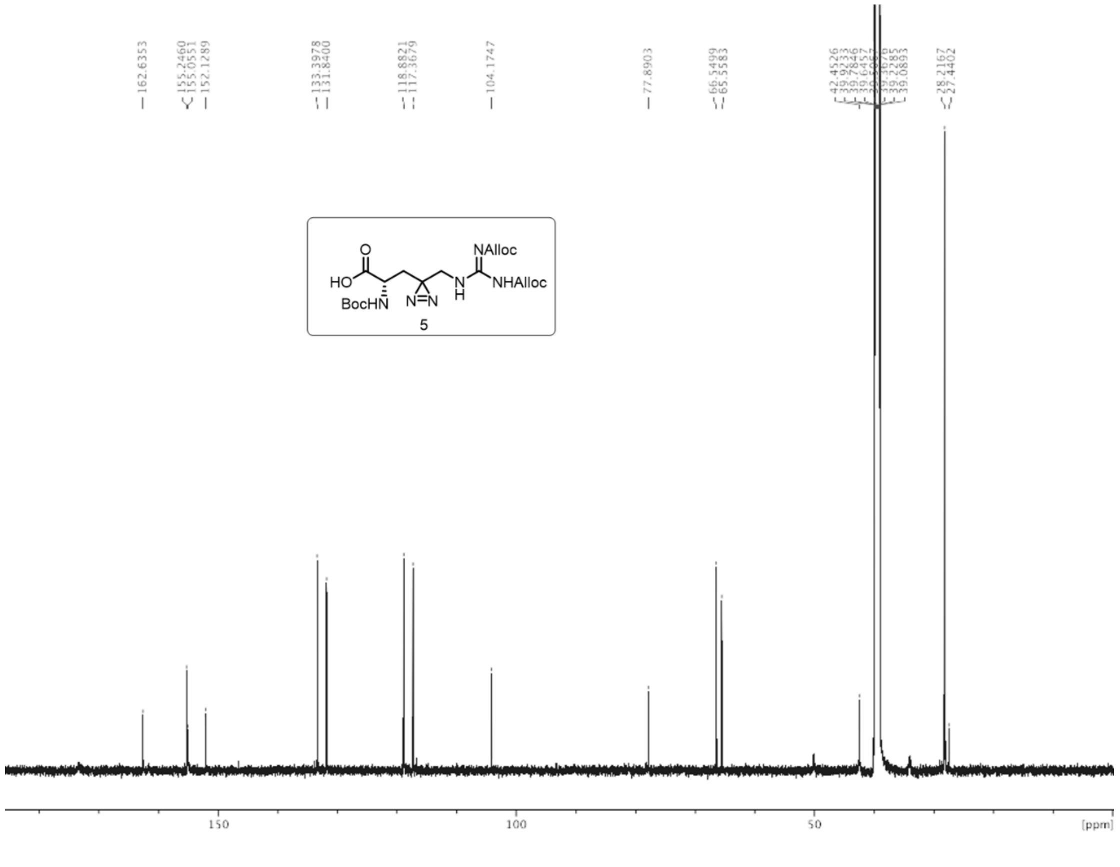
NAlloc
HO
NHAlloc
BocHN
N=N
H
5
150
100
50
[ppm]

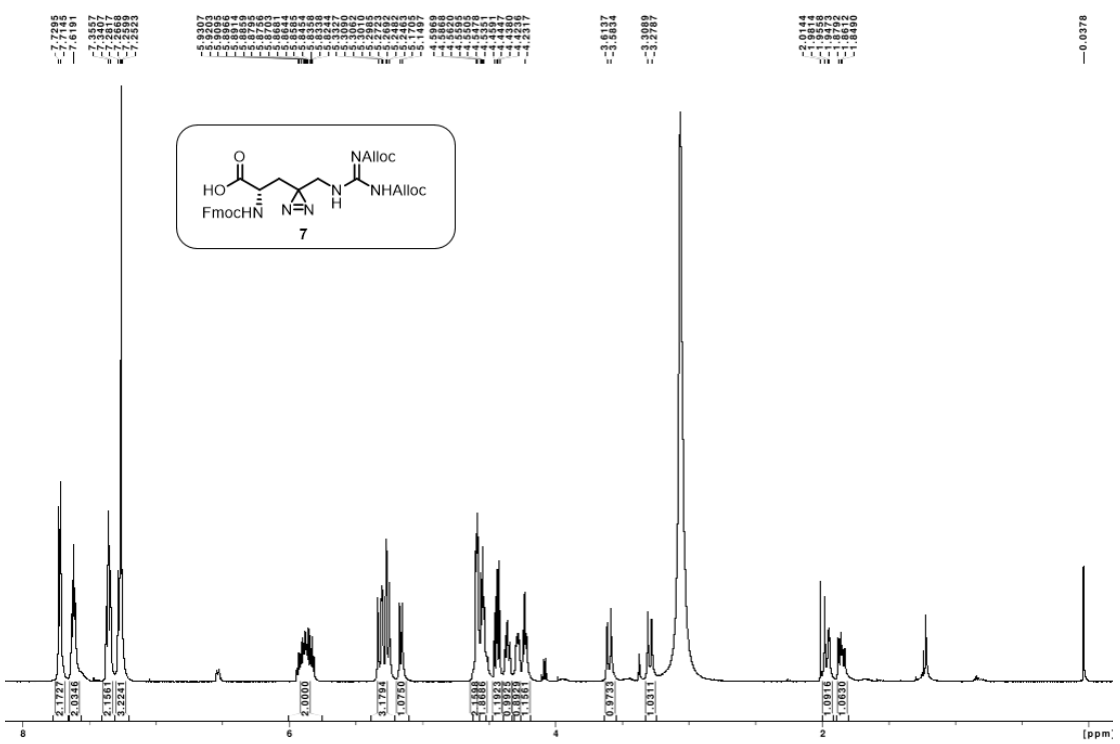

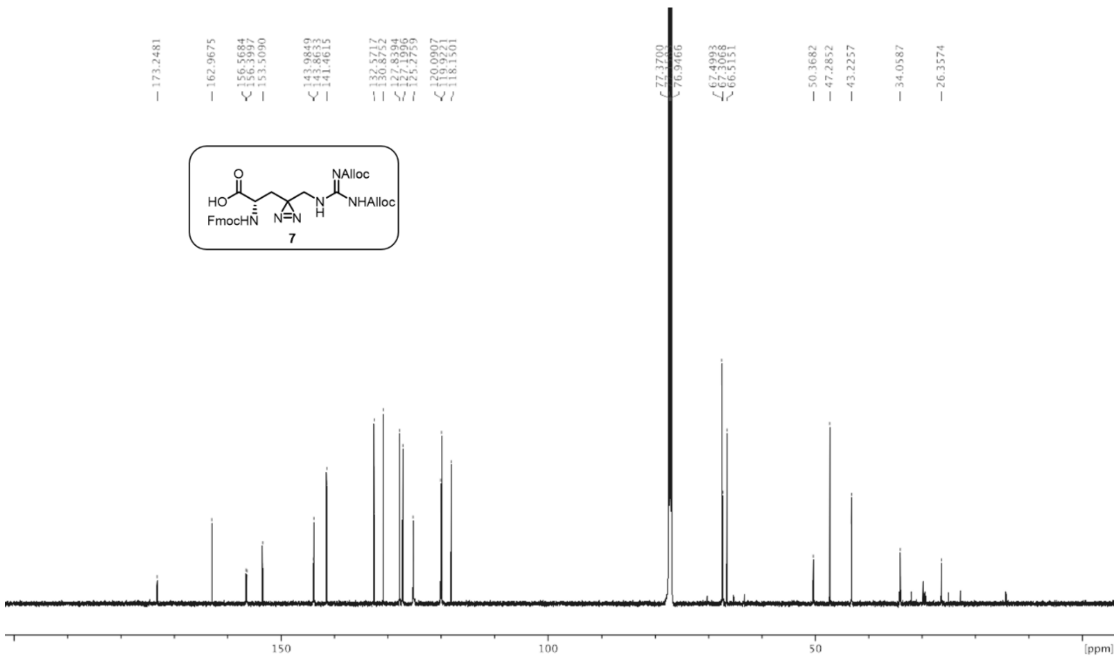

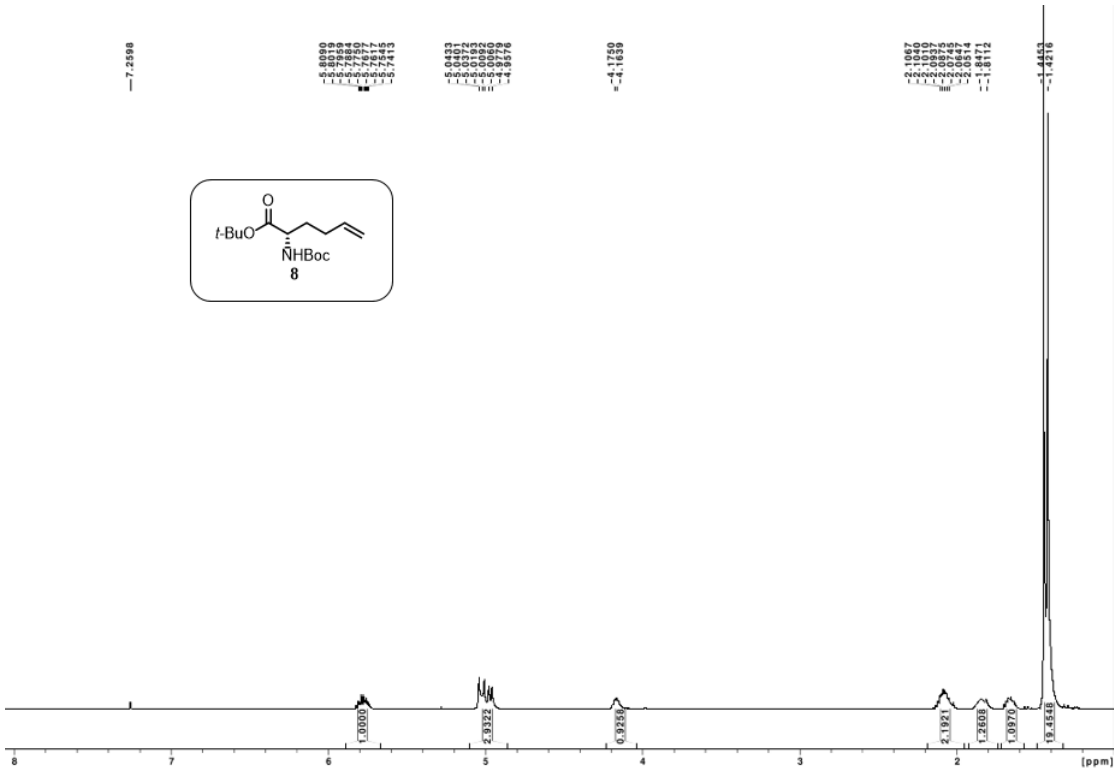
t-BuO
NHBoc
8
[ppm]

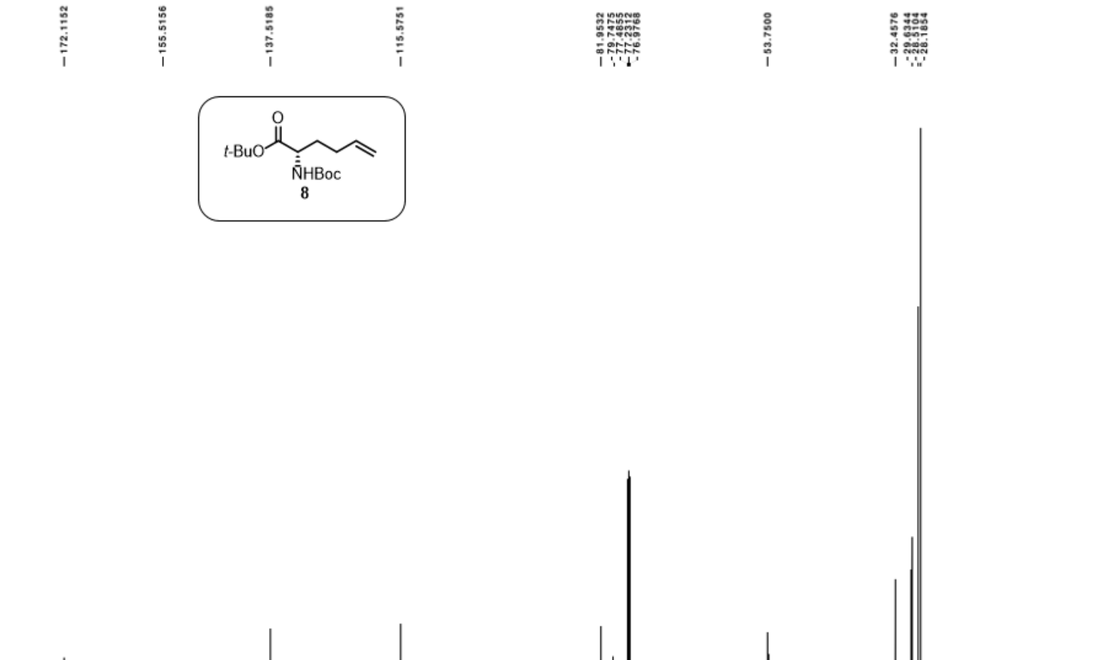
t-BuO
NHBoc
8
[ppm

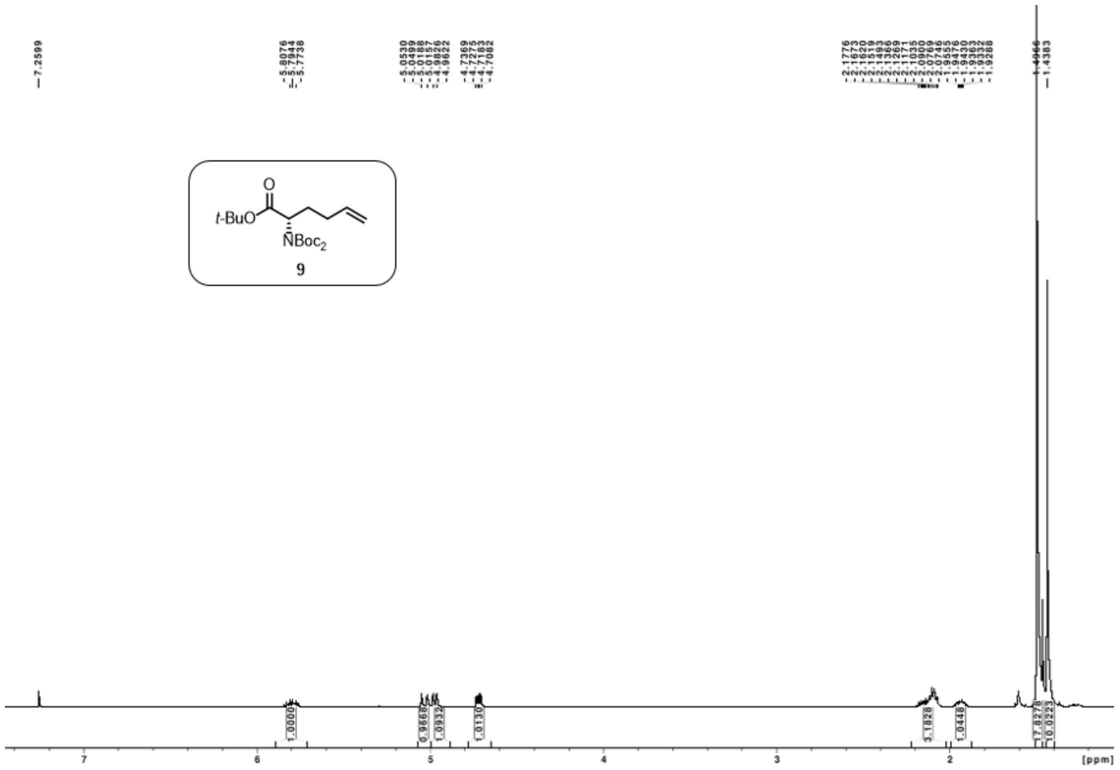

7.2599
1.4966
1.4383
t-BuO
O
NBoc2
9
[ppm]

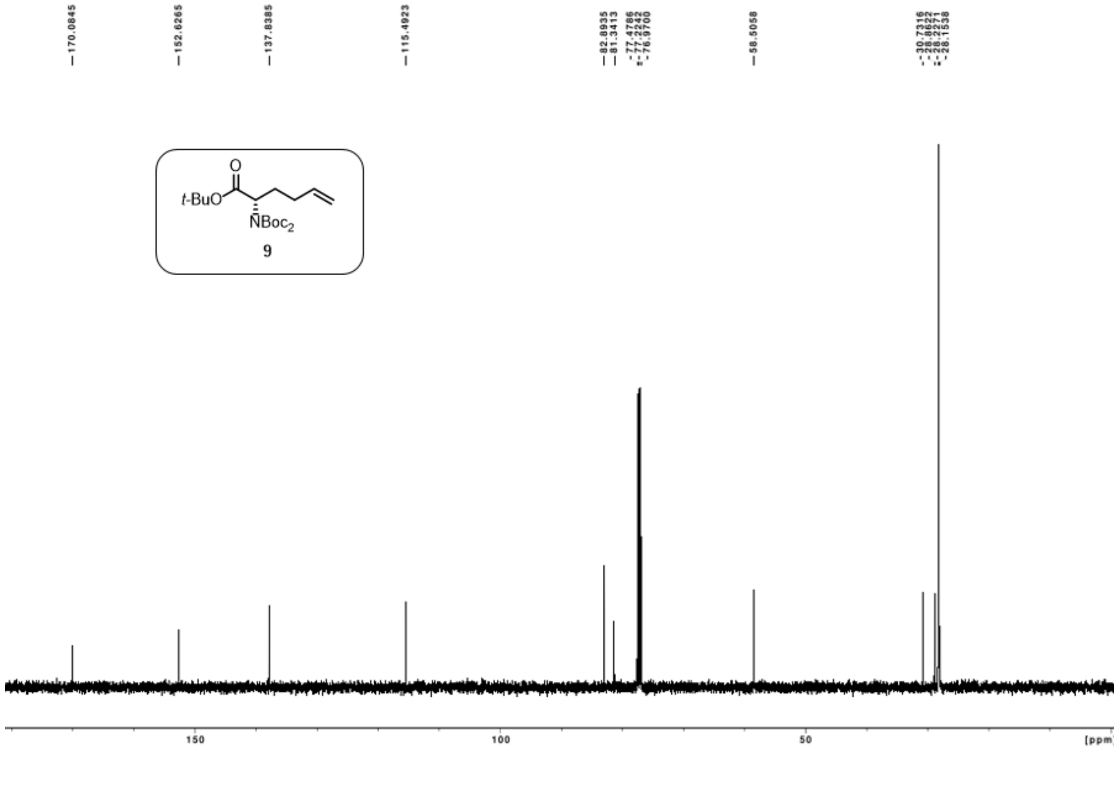

170.0845
152.6265
137.8385
115.4923
82.8935
81.3413
58.5058
t-BuO
O
NBoc2
9
150
100
50
[ppm]

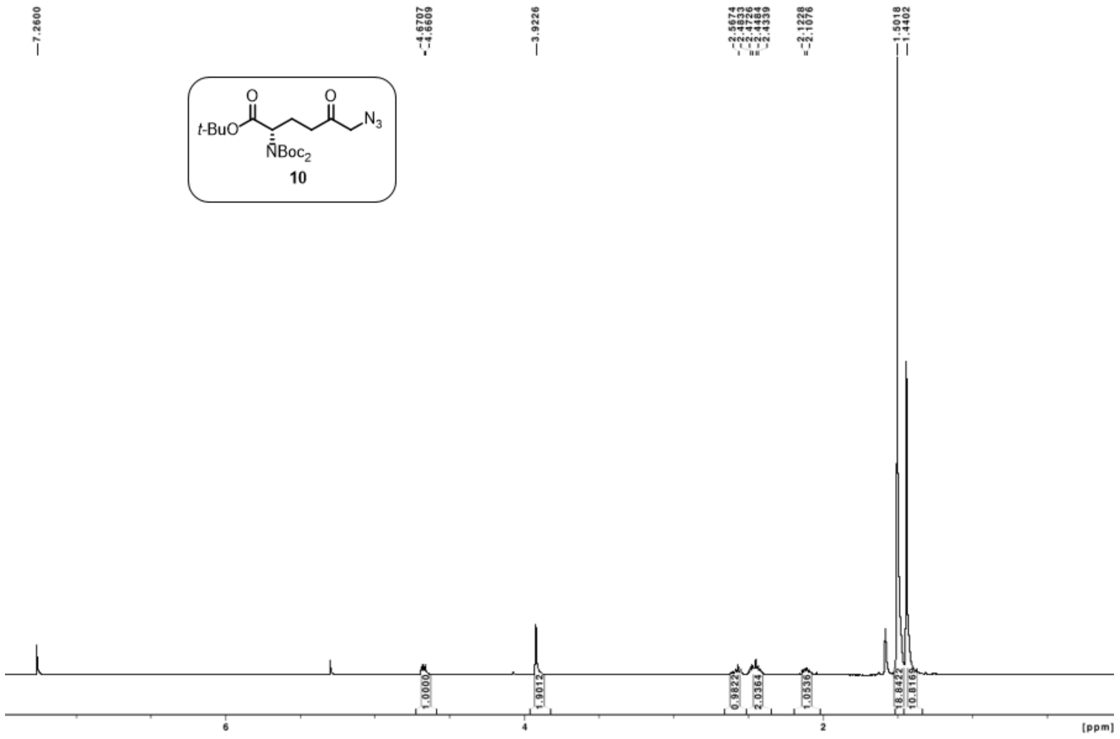

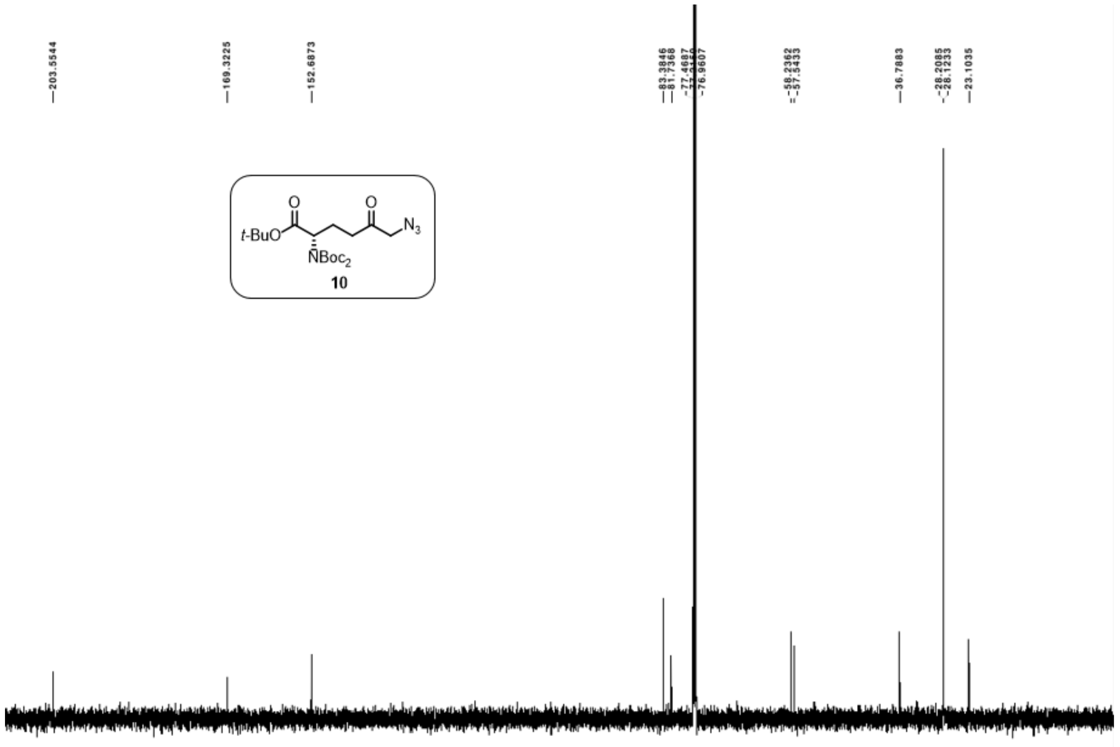

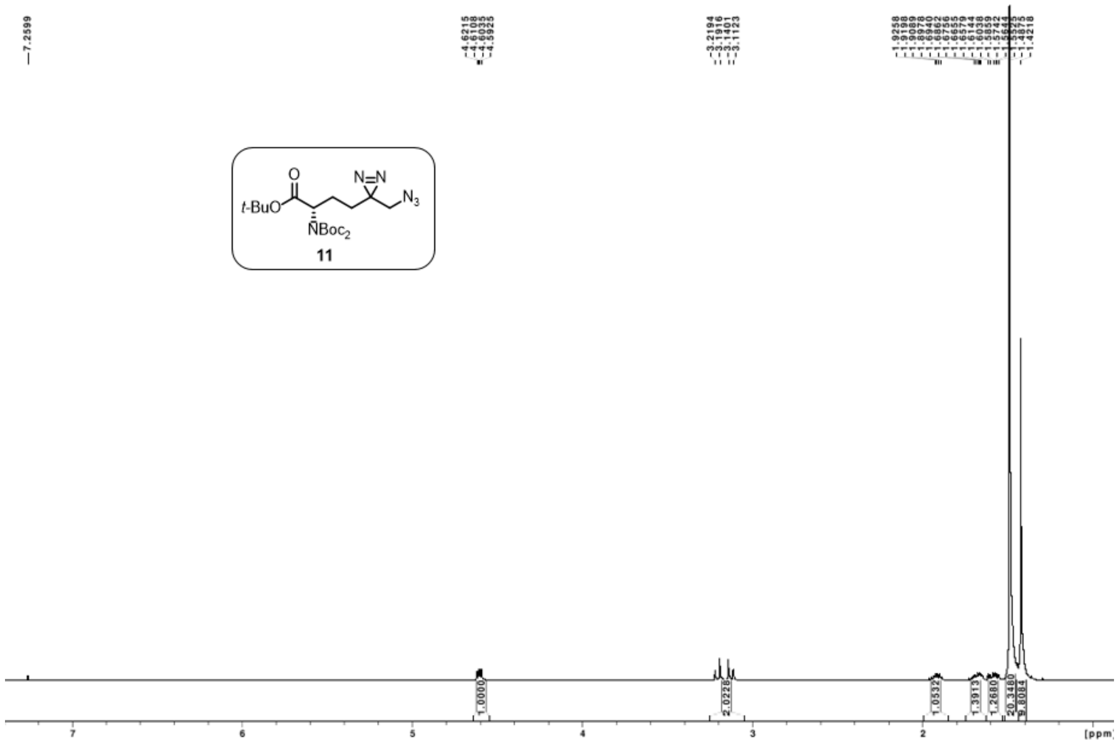
7.2599
t-BuO
NBoc2
N3
11
[ppm]

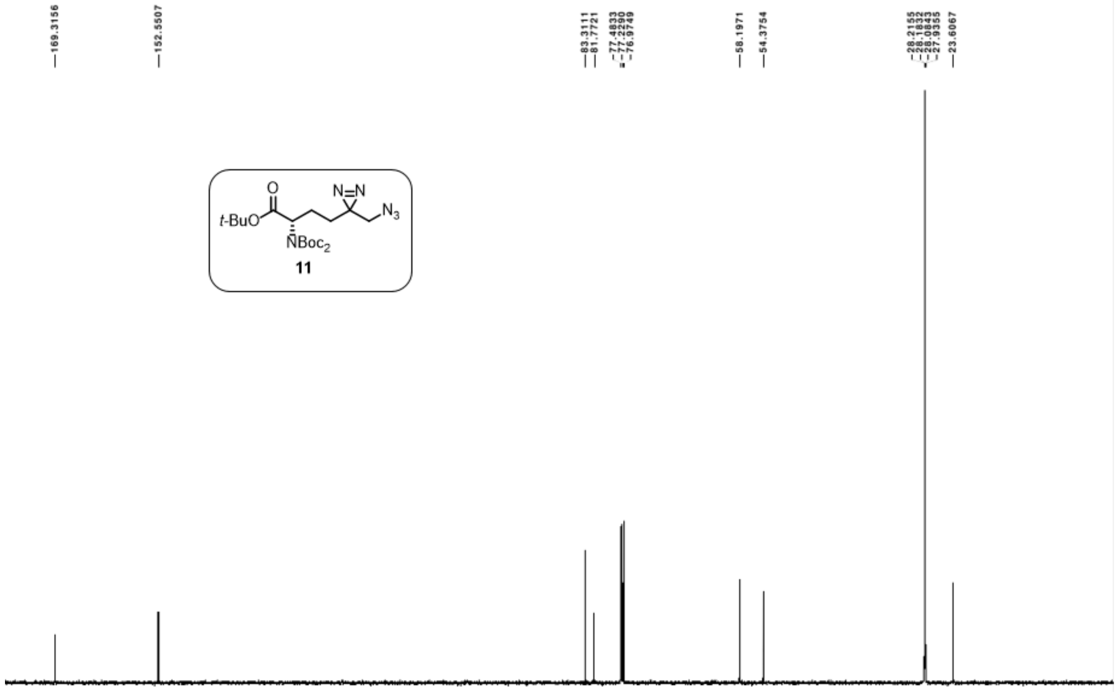
169.3156
152.5507
83.3111
81.7721
77.4833
77.2290
76.9749
58.1971
54.3754
28.2155
28.1832
28.0843
27.9355
23.6067
t-BuO
NBoc2
N3
11
[ppm]

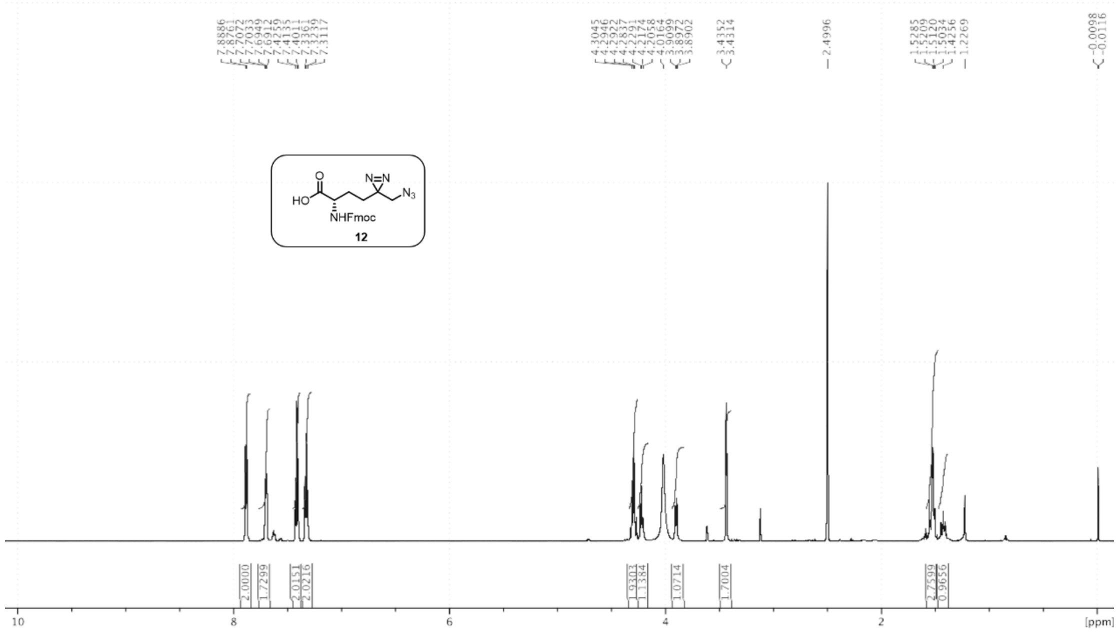
12
[ppm]

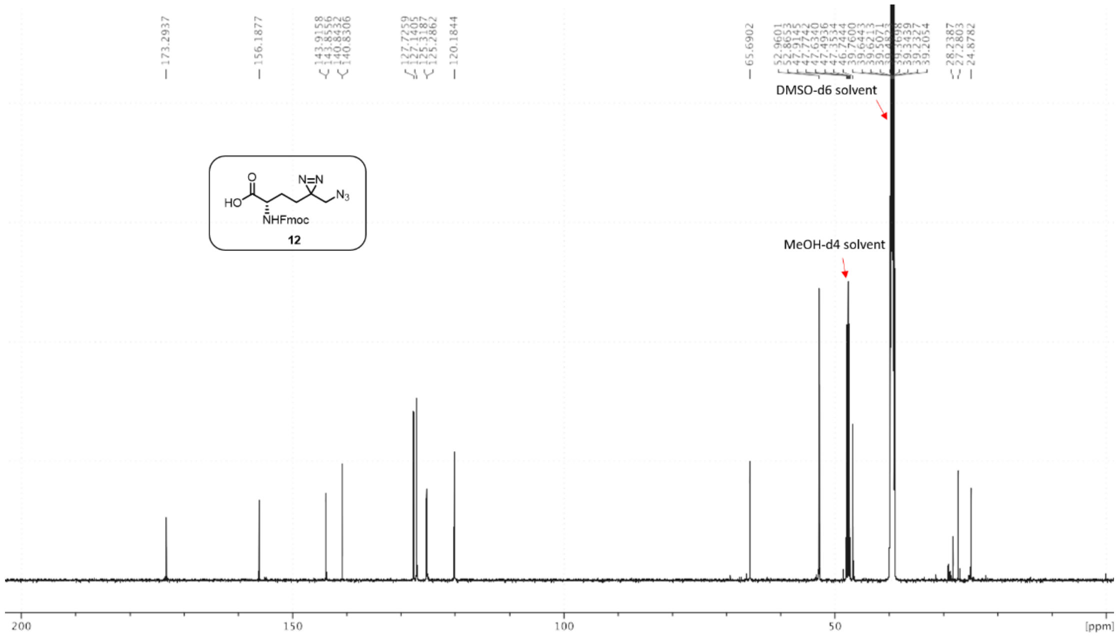
DMSO-d6 solvent
MeOH-d4 solvent
12
[ppm]

MS data

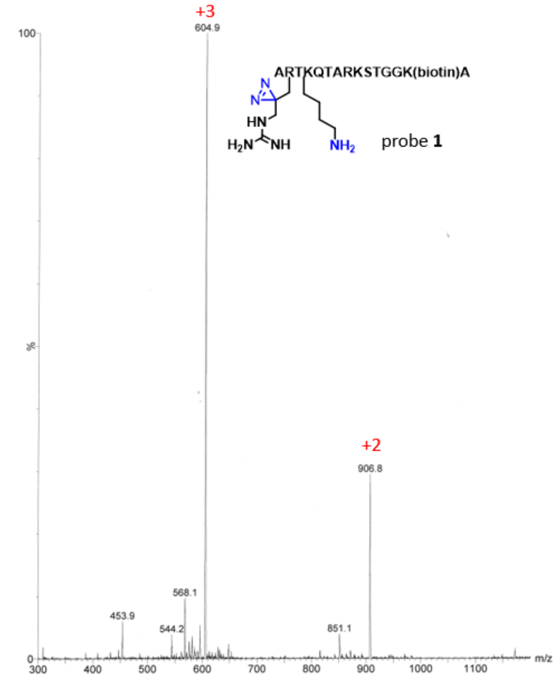
+3
604.9
ARTKQTARKSTGGK(biotin)A
probe 1
+2
906.8
568.1
453.9
544.2
851.1
m/z

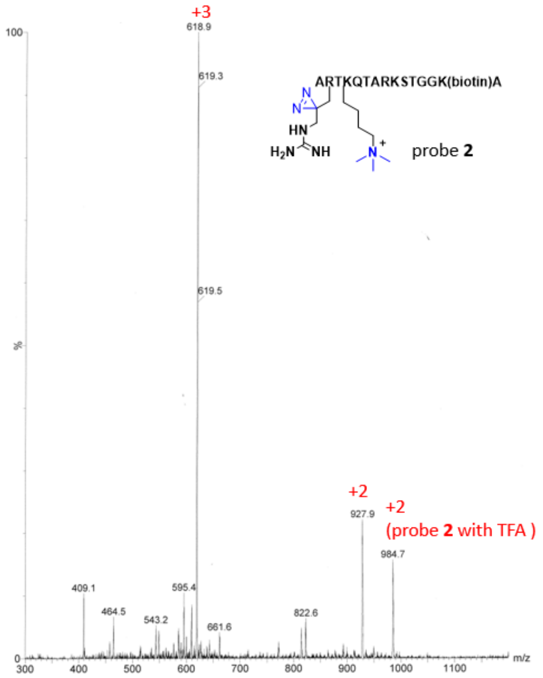
+3
618.9
619.3
ARTKQTARKSTGGK(biotin)A
probe 2
619.5
+2
927.9
+2
(probe 2 with TFA)
984.7
409.1
595.4
464.5
543.2
661.6
822.6
m/z

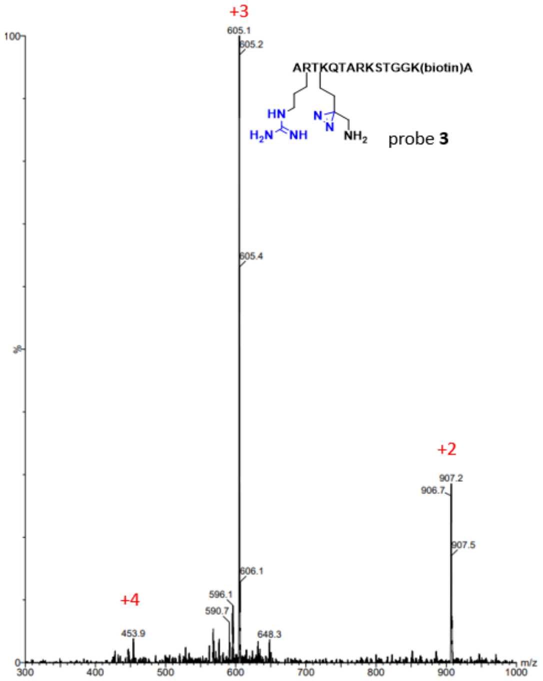
+3
605.1
605.2
ARTKQTARKSTGGK(biotin)A
probe 3
605.4
+2
907.2
906.7
907.5
606.1
+4
596.1
590.7
453.9
648.3
m/z

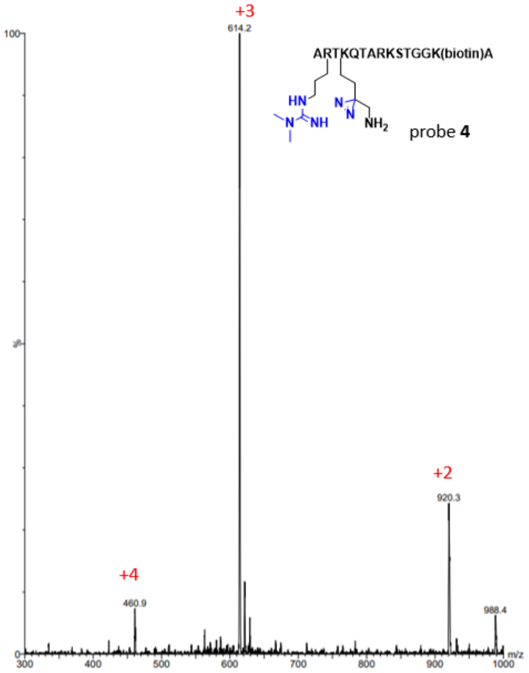
+3
614.2
ARTKQTARKSTGGK(biotin)A
probe 4
+2
920.3
+4
460.9
988.4
m/z

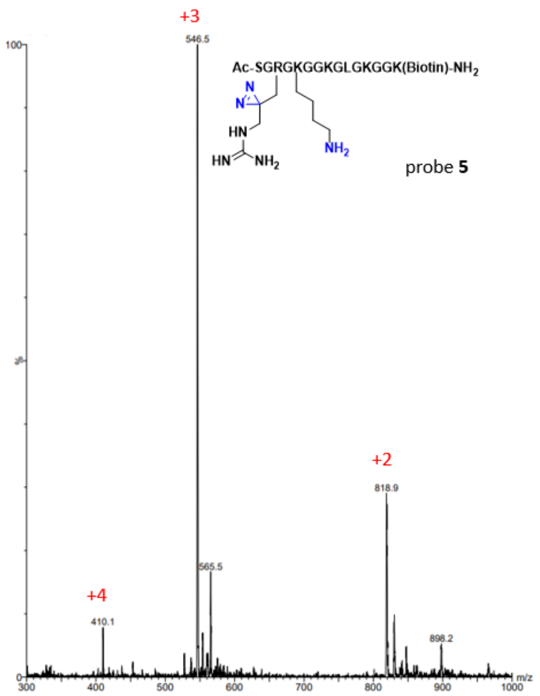
Ac-SGRGKGGKGLGKGGK(Biotin)-NH2
probe 5
+3
546.5
+2
818.9
+4
410.1
565.5
898.2
m/z

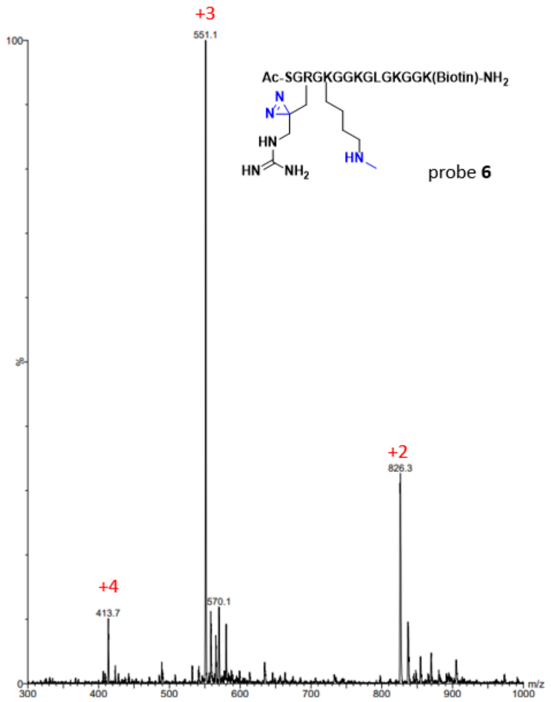
Ac-SGRGKGGKGLGKGGK(Biotin)-NH2
probe 6
+3
551.1
+2
826.3
+4
413.7
570.1
m/z

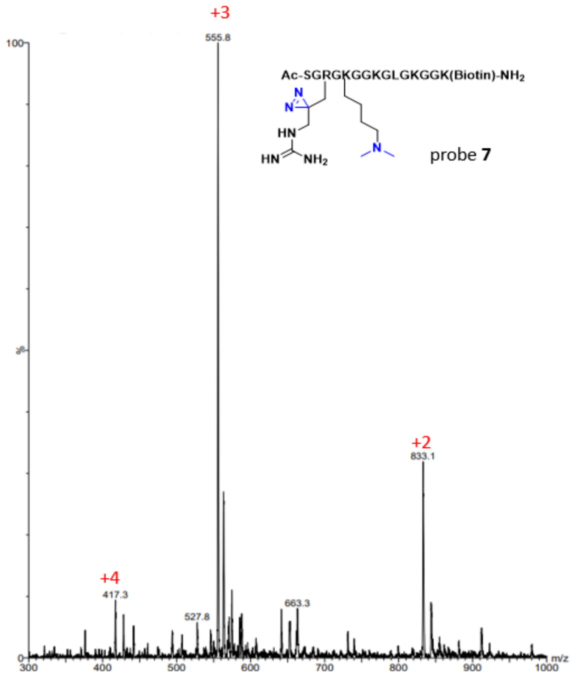
Ac-SGRGKGGKGLGKGGK(Biotin)-NH2
probe 7
+3
555.8
+2
833.1
+4
417.3
527.8
663.3
m/z

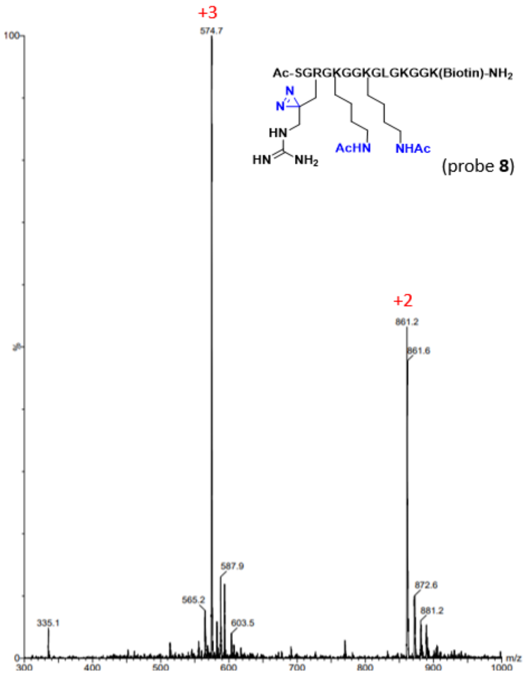
Ac-SGRGKGGKGLGKGGK(Biotin)-NH2
AcHN
NHAc
(probe 8)
+3
574.7
+2
861.2
861.6
587.9
565.2
603.5
872.6
881.2
335.1
m/z

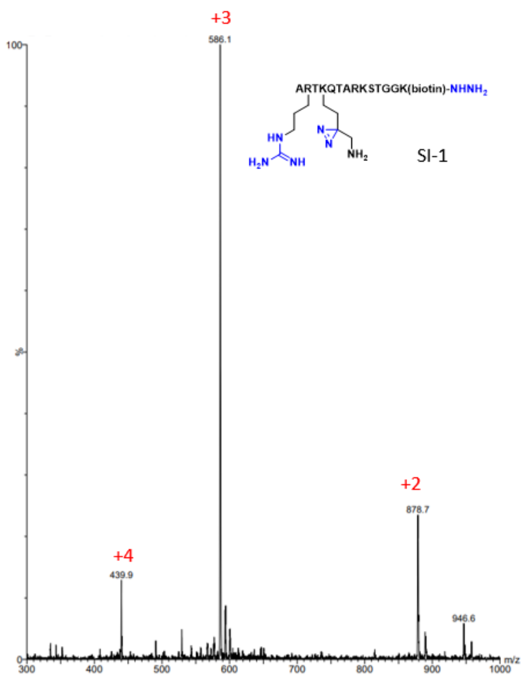
ARTKQTARKSTGGK(biotin)-NHNH2
SI-1
+3
586.1
+2
878.7
+4
439.9
946.6
m/z

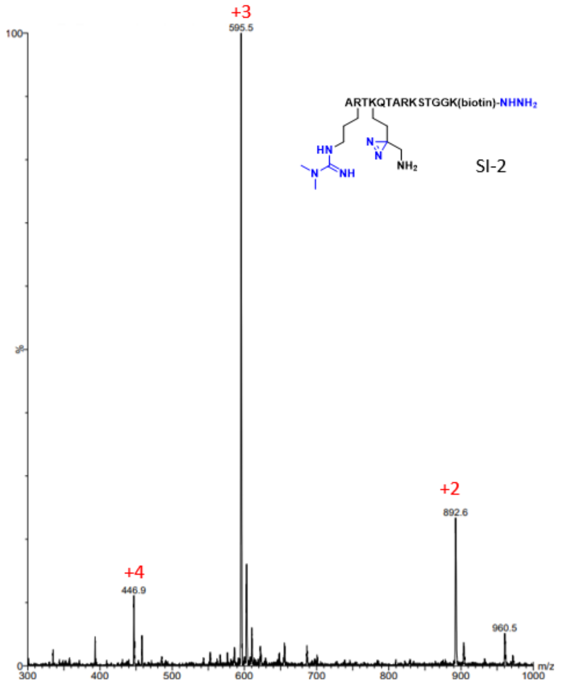
ARTKQTARKSTGGK(biotin)-NHNH2
SI-2
+3
595.5
+2
892.6
+4
446.9
960.5
m/z

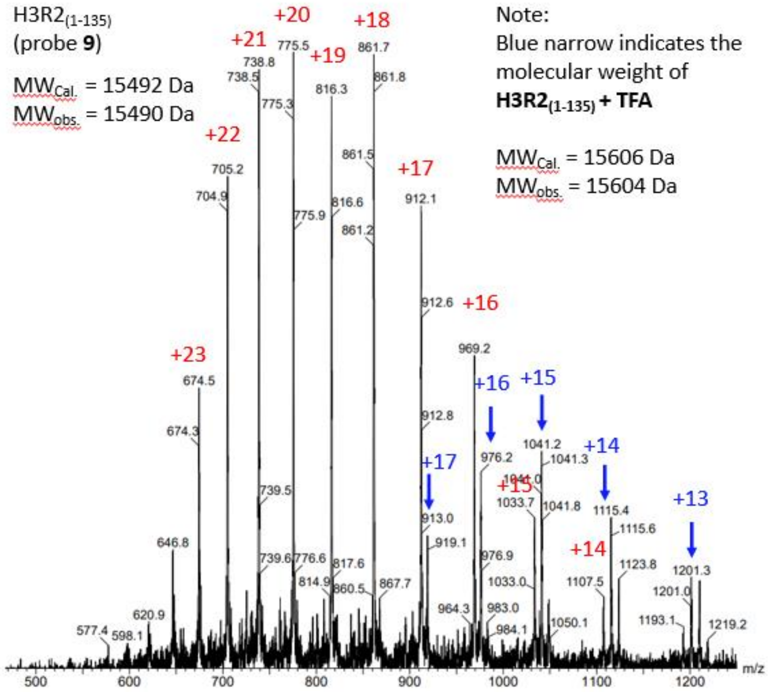
H3R2(1-135)
(probe 9)
MWCal. = 15492 Da
MWobs. = 15490 Da
Note:
Blue narrow indicates the molecular weight of
H3R2(1-135) + TFA
MWCal. = 15606 Da
MWobs. = 15604 Da
+23
+22
+21
+20
+19
+18
+17
+16
+15
+14
+13
m/z

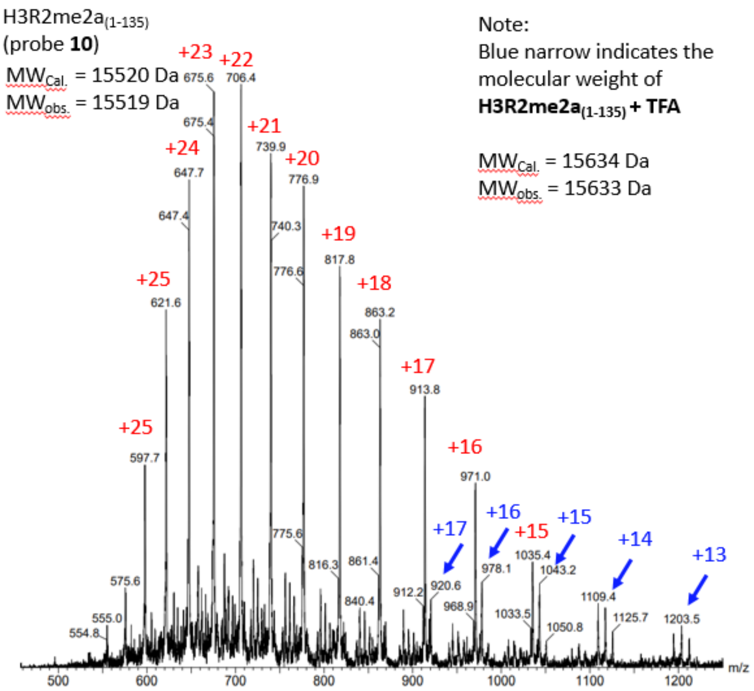
H3R2me2a(1-135)
(probe 10)
MWCal. = 15520 Da
MWobs. = 15519 Da
Note:
Blue narrow indicates the molecular weight of
H3R2me2a(1-135) + TFA
MWCal. = 15634 Da
MWobs. = 15633 Da
+25 +24 +23 +22 +21 +20 +19 +18 +17 +16 +15
+17 +16 +15 +14 +13
m/z

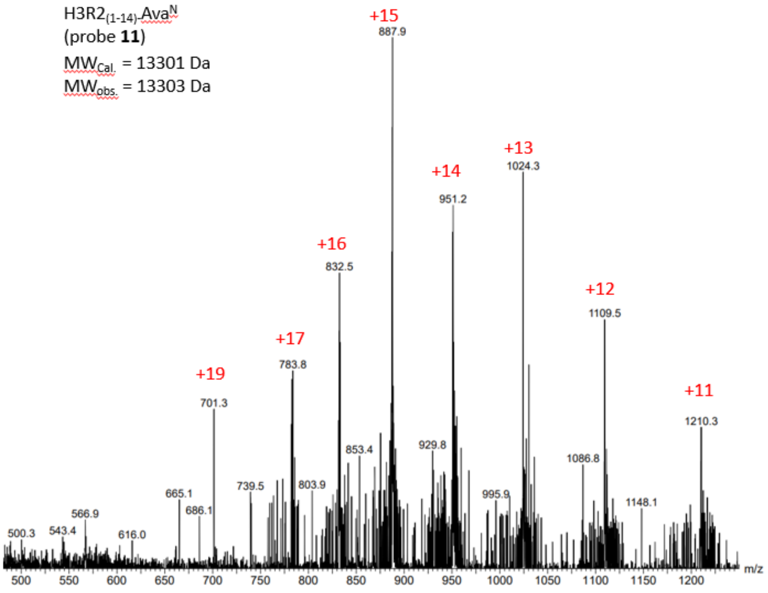
H3R2(1-14)-AvaN
(probe 11)
MWCal. = 13301 Da
MWobs. = 13303 Da
+19 +17 +16 +15 +14 +13 +12 +11
m/z

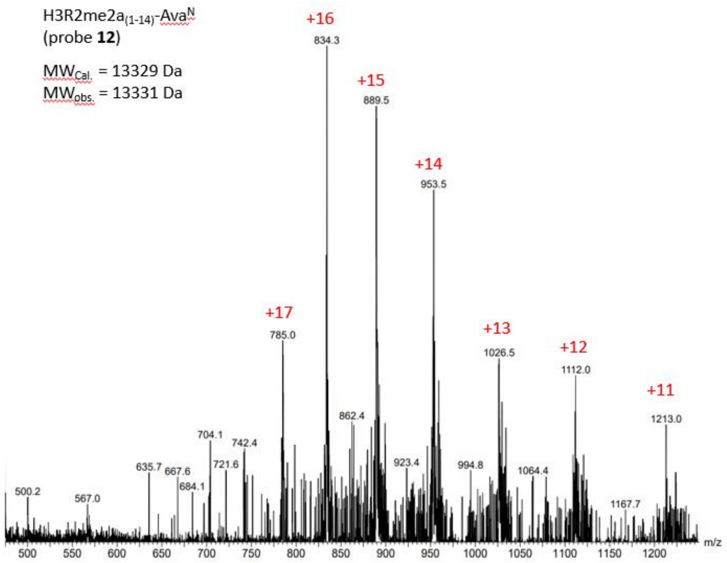

Reference

1. H. Thomanek, S. T. Schenk, E. Stein, K. Kogel, A. Schikora, W. Maison. *Org. Biomol. Chem.*, **2013**, *11*, 6994.
2. H. Konno, K. Kubo, H. Makabe, E. Toshiro, N. Hinoda, K. Nosakaa, K. Akaji. *Tetrahedron* **2007**, *63*, 9502.
3. A. Rodríguez, D. D. Miller, R. F. W. Jackson. *Org. Biomol. Chem*. **2003**, *1*, 973.
